# Design of Coibamide A Mimetics with Improved Cellular Bioactivity

**DOI:** 10.1021/acsmedchemlett.1c00591

**Published:** 2021-12-29

**Authors:** Takashi Kitamura, Rikito Suzuki, Shinsuke Inuki, Hiroaki Ohno, Kerry L. McPhail, Shinya Oishi

**Affiliations:** † Graduate School of Pharmaceutical Sciences, Kyoto University, Sakyo-ku, Kyoto 606-8501, Japan; ‡ Department of Pharmaceutical Sciences, College of Pharmacy, Oregon State University, Corvallis, Oregon 97331, United States; § Department of Medicinal Chemistry, Kyoto Pharmaceutical University, Yamashina-ku, Kyoto 607-8412, Japan

**Keywords:** apratoxin A, biphenylylalanine, coibamide A, macrocyclic peptide, Sec61, translocon

## Abstract

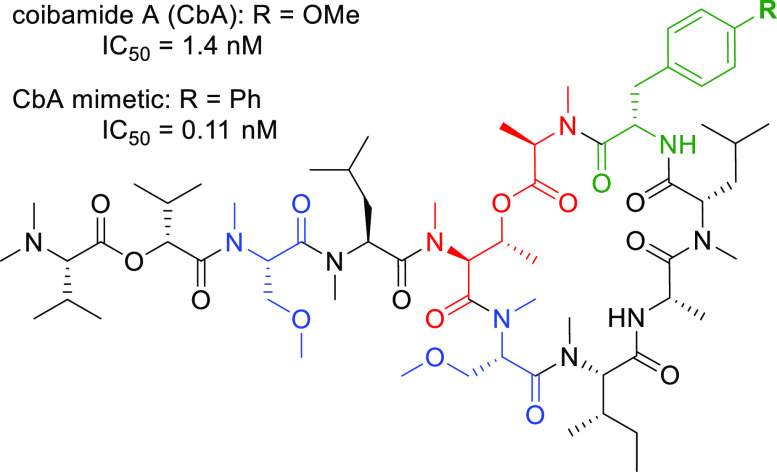

Coibamide A, a cyclic depsipeptide isolated from a Panamanian marine cyanobacterium, shows potent cytotoxic activity via the inhibition of the Sec61 translocon. We designed a coibamide A mimetic in which the ester linkage between MeThr and d-MeAla in coibamide A was replaced with an alkyl linker to provide a stable macrocyclic scaffold possessing a MeLys(Me) residue. Taking advantage of a facile solid-phase synthetic approach, an structure–activity relationship (SAR) study of the newly designed macrocyclic structure was performed, with a focus on altering the pattern of *N*-methyl substitution and amino acid configurations. Overall, the simplified macrocyclic scaffold with an alkyl linker resulted in a significantly reduced cytotoxicity. Instead, more potent coibamide A derivatives with a β-(4-biphenylyl)alanine (Bph) group were identified after the optimization of the Tyr(Me) position in the original macrocyclic scaffold of coibamide A based on the characteristic apratoxin A substructures. The similar SAR between coibamide A and apratoxin A suggests that the binding site of the Tyr(Me) side chain at the luminal end of Sec61α may be shared.

Coibamide A (CbA, **1**) is a highly *N*-methylated cyclic depsipeptide isolated from a Panamanian marine cyanobacterium (Figure 1).^1,2^ This macrocyclic natural product shows highly potent antiproliferative activity against many cell lines, with a pattern of selectivity suggestive of a distinct mechanism of action.^1^ In glioblastoma cells, CbA (**1**) induces autophagosome accumulation via a mammalian target of rapamycin (mTOR)-independent mechanism.^3^ The autophagy is mediated by autophagy-related protein 5 (ATG5), while CbA-induced apoptosis is independent of the presence of ATG5.^4^ The autophagosome clearance defects are caused by the abrogation of the autophagosome-lysosome fusion process via the impaired glycosylation of lysosomal membrane proteins LAMP1 and LAMP2.^5^ Cellular treatment of CbA (**1**) also prevents the extracellular secretion of vascular endothelial growth factor A (VEGFA) as well as the expression of vascular endothelial growth factor receptor 2 (VEGFR2) and epidermal growth factor receptors (EGFR, HER2, and HER3).^6,7^ Our recent investigation using coibamide photoaffinity probes demonstrated that CbA (**1**) directly binds to the α-subunit of a Sec61 translocon (Sec61α) to prevent the channel function of Sec61.^8^

**Figure 1:**
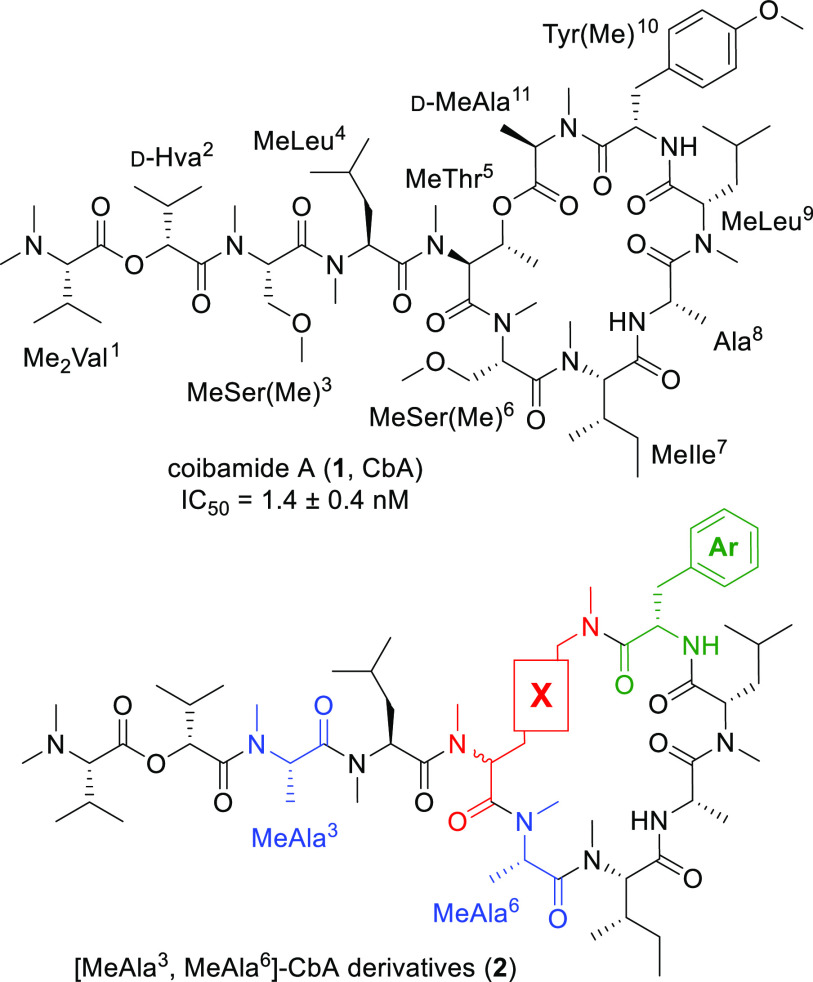
Structure of coibamide A and our plan for the SAR study.

The Sec61 translocon is a component of the protein translocation machinery for the co- and post-translational transport of secreted and transmembrane proteins into the endoplasmic reticulum.^9,10^ Because the Sec61 channel-mediated translocation of regulatory and pathogenetic proteins, such as adhesion molecules and viral proteins, is involved in the pathological process, Sec61 is a potential molecular target for anticancer and anti-infective agents.^11,12^ To date, there have been several Sec61 inhibitors reported,^13,14^ including apratoxin A,^15,16^ decatransin,^17^ eeyarestatin I,^18,19^ HUN-7293/pestahivin,^20−22^ ipomoeassin F,^23^ and mycolactone A and B^24^ (Figure S1). For the application of these promising inhibitors to drug discovery, considerable efforts have been devoted to their medicinal chemistry studies.^25−31^ On the basis of these insights into Sec61 inhibitors, we investigated the structure–activity relationships (SARs) of CbA (**1**) in this study.

We designed a simplified analogue **2** in which the ester linkage between the hydroxy group of l-MeThr^5^ and carboxy group of d-MeAla^11^ in **1** was substituted with an alkyl tether (Figure 1). The resulting arrangement of MeLys(Me) at the MeThr^5^–d-MeAla^11^ moiety would provide resistance against possible degradation via the hydrolysis of the labile ester bond or the β-elimination of *O*-acyl threonine to enhance the molecular stability. Additionally, two MeSer(Me) moieties in **1** were substituted with MeAla (MeAla^3^ and MeAla^6^) because the bioactivity of the MeAla analogue was comparable to that of the parent peptide, as reported previously.^28^ These modifications would facilitate the synthesis of a series of derivatives, especially to avoid the epimerization^32^ that is possible during couplings between the MeThr hydroxy group and *N*-methylamino acids.

Initially, we established a synthetic route to [MeAla^3^, MeLys(Me)^5^, MeAla^6^]-CbA (**2a**, Scheme 1). The peptide sequence was assembled by Fmoc-based solid-phase peptide synthesis (Fmoc-SPPS) using the MeLeu–(2-Cl)Trt resin **3**. 1-[Bis(dimethylamino)methylene]-1*H*-1,2,3-triazolo[4,5-*b*]pyridinium 3-oxide hexafluorophosphate (HATU)/*N*,*N*-diisopropylethylamine (DIEA) was exploited for amino acid couplings onto *N*-methylamino acids. For protection of the ε-amino group of Lys^5^ at the ring junction, orthogonal allyloxycarbonyl (Alloc) protection was employed. After the coupling of Lys(Alloc)^5^, followed by the deprotection of the Fmoc group, the resin **4** was subjected to an on-resin *N*-methylation protocol.^33^ Briefly, after the α-amino group of Lys(Alloc)^5^ was activated with an *o*-nitrobenzenesulfonyl (Ns) group, the *N*^α^-methyl group was introduced by a Mitsunobu reaction. The subsequent deprotection of the Ns group afforded the MeLys(Alloc)^5^ residue in **5**. Further couplings of the depsipeptide’s N-terminal tail (Me_2_Val^1^–d-Hva^2^–MeAla^3^–MeLeu^4^) using HATU/DIEA provided the linear peptidyl resin. Next, we proceeded to modify the MeLys(Alloc)^5^. *N*-Methylation of the MeLys ε-amine was performed by the Pd(PPh_3_)_4_/PhSiH_3_-mediated removal of the *N*^ε^-Alloc group, followed by the on-resin *N*-methylation protocol to construct the MeLys(Me)^5^ residue in resin **6**. The coupling of Tyr(Me)^10^ onto the ε-*N*-methylamino group of MeLys(Me)^5^ provided the open-chain precursor **7**. Cleavage from the resin **7**, followed by macrocyclization with EDCI/HOAt/DIEA, gave the desired cyclic peptide **2a**. As such, we developed a facile solid-phase synthesis of CbA mimetics with a MeLys(Me) moiety at the ring junction. Using a variety of commercially available materials for the components, a series of structural analogues could be obtained by the same procedure. Of note, the resulting peptide **2a** exhibited submicromolar cytotoxicity against A549 cells in an MTS assay [IC_50_ (**2a**) = 0.42 μM].

**Scheme 1:**
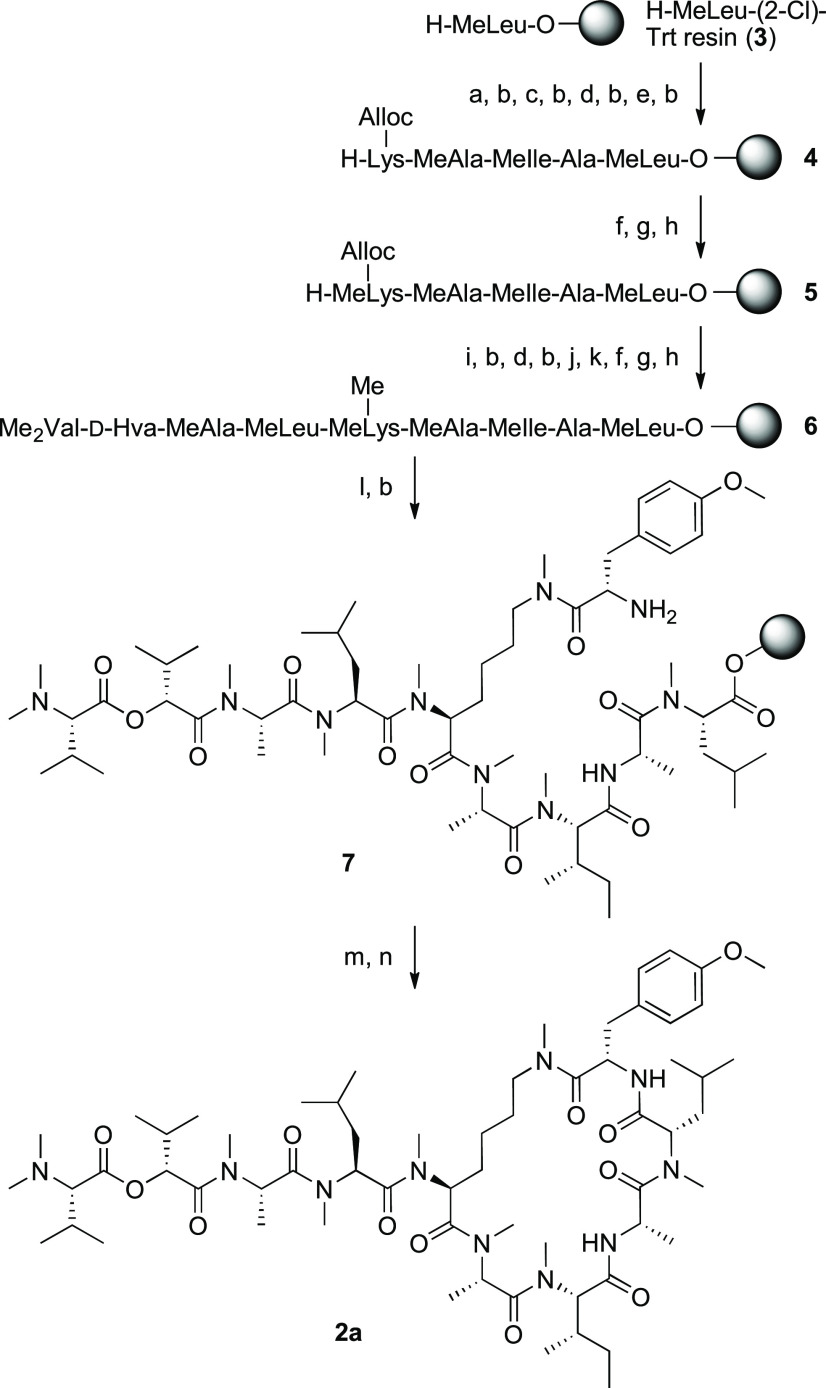
Synthesis of [MeAla^3^, MeLys(Me)^5^, MeAla^6^]-Coibamide A (**2a**) Reagents and conditions are as follows: (a) Fmoc-Ala-OH·H_2_O, HATU, DIEA, DMF, 40 °C; (b) 20% piperidine/DMF, rt; (c) Fmoc-MeIle-OH, HOBt·H_2_O, DIC, DMF, 40 °C; (d) Fmoc-MeAla-OH, HATU, DIEA, DMF, 40 °C; (e) Fmoc-Lys(Alloc)-OH, HATU, DIEA, DMF, 40 °C; (f) NsCl, 2,4,6-collidine, NMP, rt; (g) Ph_3_P, DEAD, MeOH, THF, rt; (h) 2-mercaptoethanol, DBU, NMP, rt; (i) Fmoc-MeLeu-OH, HATU, DIEA, DMF, 40 °C; (j) Me_2_Val-d-Hva-OH, HATU, DIEA, NMP, 40 °C; (k) Pd(PPh_3_)_4_, PhSiH_3_, CH_2_Cl_2_, rt; (l) Fmoc-Tyr(Me)-OH, HATU, DIEA, NMP, 40 °C; (m) 30% HFIP/CH_2_Cl_2_, rt; (n) EDCI·HCl, HOAt, DIEA, DMF, 0 °C to rt.

Next, we investigated ring junction SARs for these CbA mimetics. Because substitution of the ester linkage with an ethylene tether in **2a** would alter the global conformations of the cyclic substructure of **1**, we attempted optimization at the MeLys(Me)^5^ moiety in **2a** via modifying the tether length, the amino acid configuration, and the presence or absence of the *N*-methyl group. For this purpose, we substituted several lysine (Lys) and ornithine (Orn) moieties at the l-MeLys(Me)^5^ position of **2a** (Table 1). Inversion of the stereochemistry from l-MeLys(Me)^5^ led to a 19-fold decrease in the cytotoxicity compared with that of peptide **2a** [IC_50_ (**2b**) = 8.3 μM]. Similarly, truncation of the tether length to l- or d-MeOrn(Me) resulted in a moderate reduction or loss of cytotoxicity [IC_50_ (**2c**) = 9.6 μM; IC_50_ (**2d**) > 10 μM]. In contrast, removing the *N*^ε^-methyl group of MeLys(Me) in **2a** (via substitution with MeLys) had less impact on the cytotoxicity [IC_50_ (**2e**) = 0.85 μM]. Among this series, peptide **2a** exhibited the most potent cytotoxicity, although it was approximately 300-times less active than the natural product **1**. Accordingly, the l-configuration and side chain C4-tether of l-MeLys(Me)^5^ in **2a** enabled the macrocycle to adopt favorable conformations, while the presence or absence of the *N*^ε^-methyl group was less significant.

**Table 1:**
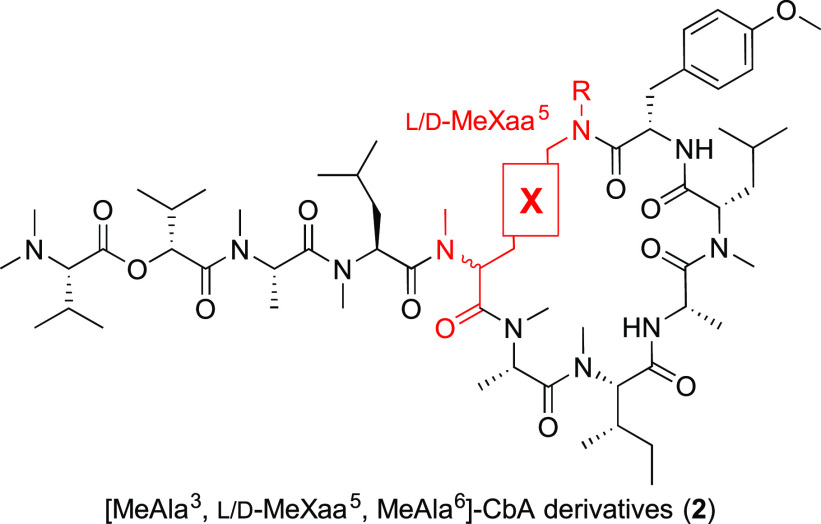
Structure–Activity Relationships of Analogues with Lys and Orn in Place of the Ester Linkage of CbA

peptide	l- or d-MeXaa^5^	IC_50_ (μM)^a^
**2a**	l-MeLys(Me)	0.42 ± 0.03
**2b**	d-MeLys(Me)	8.3 ± 2.2
**2c**	l-MeOrn(Me)	9.6 ± 3.2
**2d**	d-MeOrn(Me)	>10
**2e**	l-MeLys	0.85 ± 0.02
**2f**	d-MeLys	>10
**2g**	l-MeOrn	>10
**2h**	d-MeOrn	>10

a IC_50_ values are the concentrations for 50% growth inhibition of A549 cells (*n* = 3).

To obtain further SAR information on the backbone conformations of **2a**, we then designed and synthesized a series of derivatives substituted with an *N*-demethyl or d-amino acid (Table 2). Removal of the *N*^α^-methyl group from MeAla,^3^ MeLeu^4^, and MeLys(Me)^5^ in the N-terminal chain resulted in decreased cytotoxicities [IC_50_ (**8a**) = 2.2 μM; IC_50_ (**8b**) = 3.4 μM; IC_50_ (**8c**) = 6.4 μM], suggesting that *N*-methylation induces structural organization in the N-terminal chain, which is important for biological action. Derivatives with *N*-methyl-deficient modifications for MeAla^6^, MeIle^7^, or MeLeu^9^ in the cyclic substructure exhibited cytotoxicities nine-fold or more lower compared with that of **2a** [IC_50_ (**8d**) = 7.5 μM; IC_50_ (**8e**) > 10 μM; IC_50_ (**8f**) = 3.9 μM]. In contrast to the less significant *N*^ε^-methyl group of MeLys(Me)^5^ in **2a**, all *N*^α^-methyl groups on the macrocycle backbone of **1** were indispensable for its potent biological activity.

**Table 2:**
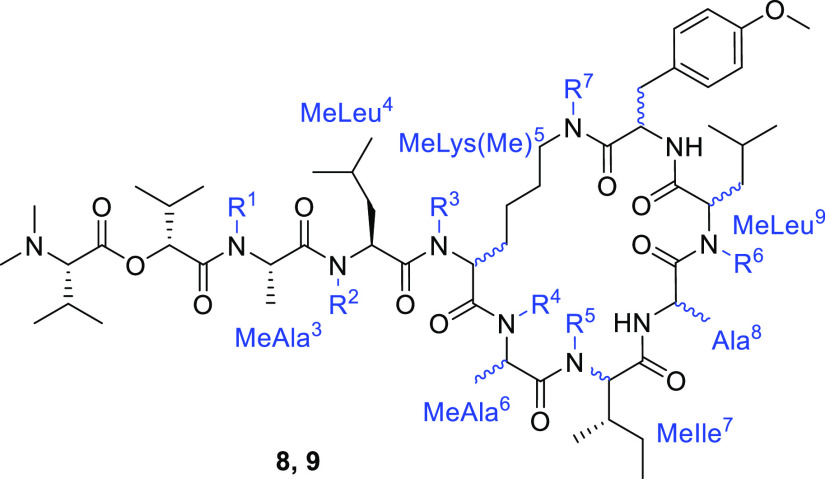
Modification of the Macrocyclic Structure by Substitution with *N*-Demethylated and d-Amino Acids

peptide	modification	IC_50_ (μM)^a^
*N*-Demethyl amino acid		
**2a**		0.42 ± 0.03
**8a**	Ala^3^	2.2 ± 0.6
**8b**	Leu^4^	3.4 ± 0.6
**8c**	Lys(Me)^5^	6.4 ± 1.7
**8d**	Ala^6^	7.5 ± 3.2
**8e**	Ile^7^	>10
**8f**	Leu^9^	3.9 ± 0.4
**2e**	MeLys^5^	0.85 ± 0.02
d-Amino acid		
**2b**	d-MeLys(Me)^5^	8.3 ± 2.2
**9a**	d-MeAla^6^	>10
**9b**	d-*allo*-MeIle^7^	>10
**9c**	d-Ala^8^	>10
**9d**	d-MeLeu^9^	2.6 ± 0.9
**9e**	d-Tyr(Me)^10^	>10

a IC_50_ values are the concentrations for 50% growth inhibition of A549 cells (*n* = 3).

We also assessed the cytotoxicities of epimers of peptide **2a** in which one of the component amino acids in the macrocycle was replaced with a d-amino acid (Table 2). Among these, the d-MeLys(Me)^5^ isomer **2b** and d-MeLeu^9^ isomer **9d** exhibited moderate cytotoxicities [IC_50_ (**2b**) = 8.3 μM; IC_50_ (**9d**) = 2.6 μM]. The other epimers **9a**–**c** and **9e** showed no cytotoxicity, demonstrating that the all-l-configuration in the macrocycle of **2a** is necessary for potent bioactivity. Notably, the cytotoxicity may be attributable both to the binding affinity to the target(s) and the membrane permeability if the target(s) exists in an intracellular compartment, as is the case for the Sec61 translocon target of **1**.^8^ Considering that the permeability of cyclic peptides is highly dependent on the number and position(s) of *N*-methyl groups and d-amino acid(s),^34^ our findings provide support that the pattern of *N*-methylation and the configurations of the peptide backbone in naturally occurring **1** have been optimized over the course of molecular evolution.

With the information on a favorable backbone structure in hand, we next proceeded to optimize the aromatic amino acid at Tyr(Me)^10^ in **2a**. To gain clues for designing the peptides, we focused on a substructure in apratoxin A (Figure S1), which is also a depsipeptide inhibitor that targets Sec61α.^15,16^ Similar to **1**, apratoxin A contains l-Tyr(Me) as the sole aromatic amino acid, which is indispensable for the bioactivity.^35^ In the previous SAR study, replacing l-Tyr(Me) in apratoxin A with l-β-(4-biphenylyl)alanine (Bph) led to a >100-fold increase in its cytotoxicity.^27^ On the basis of this insight, we pursued the development of more potent analogues by modifying Tyr(Me)^10^ in **2a** (Table 3). For this purpose, the solid-phase synthetic protocol for **2a** was fully compatible with the divergent synthesis of derivatives in which resin **6** was employed as a common substrate for further modification with various aromatic amino acids. First, a series of functional groups at the *para*-position in place of the methoxy group were investigated (**10a**–**j**). Substituting Tyr(Me) with Phe led to an approximatly 10-fold decrease in the bioactivity [IC_50_ (**10a**) = 4.0 μM]. Derivatives with a Phe(4-NO_2_), Phe(4-CN), or Tyr(*t*-Bu) group showed somewhat less potent cytotoxicities than **2a** [IC_50_ (**10b**) = 1.1 μM; IC_50_ (**10d**) = 1.5 μM; IC_50_ (**10h**) = 1.0 μM], whereas other derivatives exhibited the same level of cytotoxicity as **2a** [IC_50_ (**10c**) = 0.37 μM; IC_50_ (**10e**) = 0.38 μM; IC_50_ (**10f**) = 0.71 μM; IC_50_ (**10g**) = 0.61 μM; IC_50_ (**10i**) = 0.32 μM]. As expected, Bph-containing **10j** exhibited a seven-fold more potent cytotoxicity than **2a** [IC_50_ (**10j**) = 0.060 μM]. We further designed and synthesized derivatives (**10k**–**q**) with a variable aromatic amino acid at the Tyr(Me)^10^ position of **2a**. Pyridine-containing derivatives were inactive (**10k**–**m**). Modification with 1,2,3,4-tetrahydroisoquinoline-3-carboxylic acid (Tic, **10o**), a conformationally restricted analogue of Phe, or MePhe also led to a loss of bioactivity (**10n**), implying that *N*-alkylation at the Tyr(Me)^10^ position may unfavorably alter the conformations of the macrocycle. Interestingly, substitution with β-(1-naphthyl)alanine (1-Nal) led to decreased cytotoxicity [IC_50_ (**10p**) = 4.8 μM], while that with β-(2-naphthyl)alanine (2-Nal) resulted in a slightly improved potency [IC_50_ (**10q**) = 0.28 μM]. These observations suggest that the substituent at the *para*-position of the aromatic ring in Tyr(Me)^10^ significantly influences the cytotoxicity.

**Table 3:**
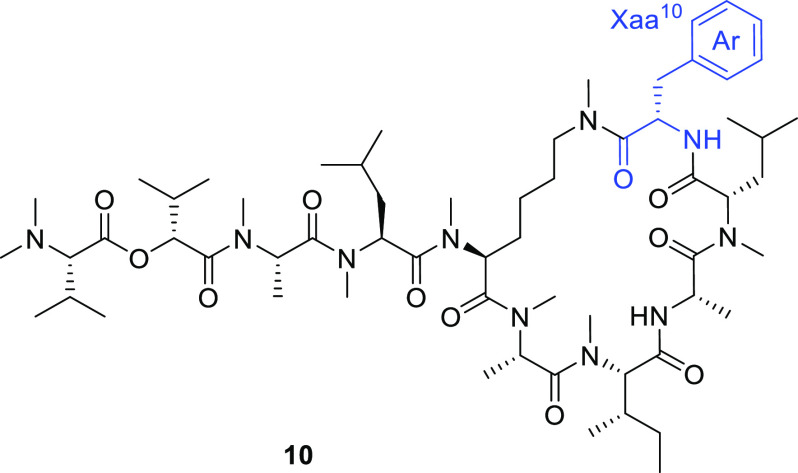
Structure–Activity Relationships of Analogues with Aromatic Amino Acids Substituted at the Tyr(Me)^10^ Moiety of CbA

peptide	Xaa^10^^a^	IC_50_ (μM)^b^
**2a**	Tyr(Me) [Phe(4-OMe)]	0.42 ± 0.03
**10a**	Phe	4.0 ± 0.8
**10b**	Phe(4-NO_2_)	1.1 ± 0.2
**10c**	Phe(4-CF_3_)	0.37 ± 0.07
**10d**	Phe(4-CN)	1.5 ± 0.5
**10e**	Phe(4-N_3_)	0.38 ± 0.05
**10f**	Phe(4-Cl)	0.71 ± 0.19
**10g**	Phe(4-*t*-Bu)	0.61 ± 0.21
**10h**	Phe(4-O*t*-Bu) [Tyr(*t*-Bu)]	1.0 ± 0.1
**10i**	Phe(4-OCF_3_) [Tyr(CF_3_)]	0.32 ± 0.03
**10j**	Bph [Phe(4-Ph)]	0.060 ± 0.016
**10k**	2-Pal	>10
**10l**	3-Pal	>10
**10m**	4-Pal	>10
**10n**	MePhe	>10
**10o**	Tic	>10
**10p**	1-Nal	4.8 ± 0.5
**10q**	2-Nal	0.28 ± 0.03

a 2-Pal, β-(2-pyridyl)alanine; 3-Pal, β-(3-pyridyl)alanine; 4-Pal, β-(4-pyridyl)alanine; Tic, 1,2,3,4-tetrahydroisoquinoline-3-carboxylic acid; 1-Nal, β-(1-naphthyl)alanine; 2-Nal, β-(2-naphthyl)alanine; Bph, β-(4-biphenylyl)alanine.

b IC_50_ values are the concentrations for 50% growth inhibition of A549 cells (*n* = 3).

Considering the enhanced potency of the Bph-containing mimetic **10j**, we designed Bph-containing analogues of the original CbA scaffold (**11** and **12**, Figure 2). Depsipeptides **11** and **12** were synthesized using some modifications of the previously reported procedure^28^ (see the Supporting Information). As expected, Bph-containing derivative **11** showed a cytotoxicity 12-fold more potent than that of **1** [IC_50_ (**11**) = 0.11 nM]. Similarly, peptide **12** with MeAla^3^ and MeAla^6^ modifications also exhibited a potency 5.6-fold greater than that of **1** [IC_50_ (**12**) = 0.25 nM]. The increased cytotoxicity obtained by replacing Tyr(Me) with Bph in CbA analogues was consistent with the SAR of apratoxin A analogues.^27^ This common SAR provides support that the binding pocket of Tyr(Me) at the luminal end of Sec61α would be shared between apratoxin A and CbA.

**Figure 2:**
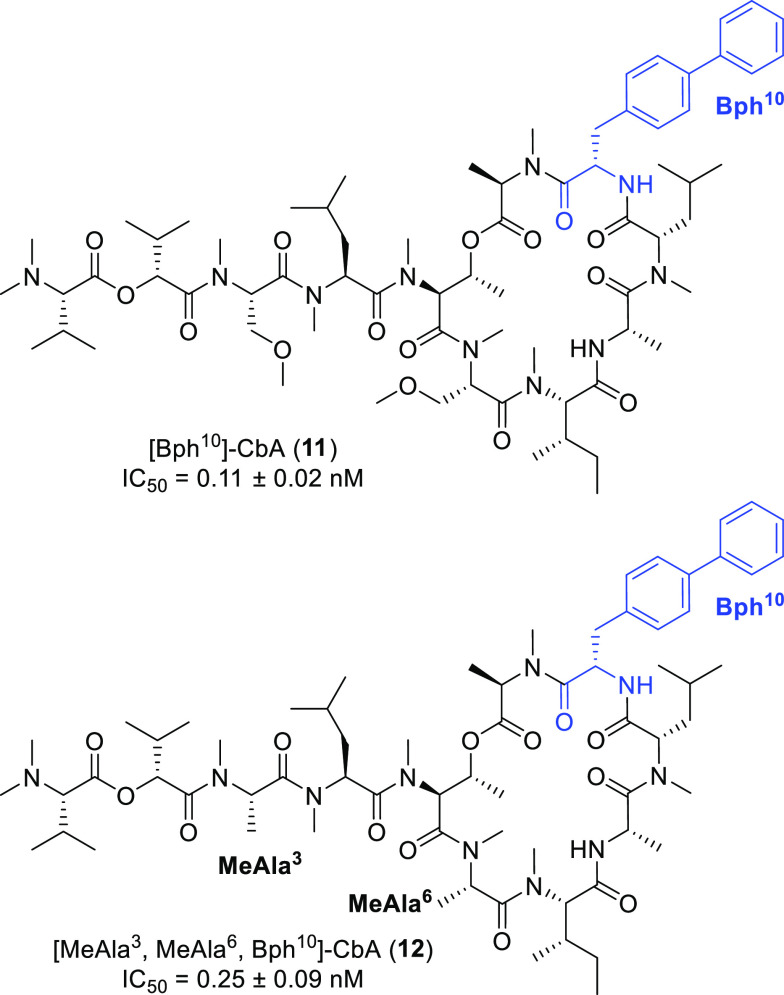
Structures of biphenylylalanine (Bph)-containing CbA analogues and their cytotoxicities. IC_50_ values are the concentrations for 50% growth inhibition of A549 cells (*n* = 3).

In summary, we designed and synthesized the CbA mimetic **2a**, which contains an alkyl linkage in place of the labile ester linkage in **1**. Taking advantage of the facile synthetic protocol established using solid-phase techniques, we investigated the SAR of macrocyclic structures of **2a**. Additionally, optimization of the aromatic amino acid in CbA was carried out based on the reported SAR data for another Sec61 inhibitory peptide, apratoxin A. The substitution of Tyr(Me)^10^ in **2a** with Bph led to significantly increased cytotoxicities, as expected. Similarly, peptides **11** and **12** with enhanced cytotoxicities were identified when the favorable Bph residue was applied to Tyr(Me)^10^ in **1** and an analogue peptide, respectively. To the best of our knowledge, this is the first report of the identification of more potent CbA analogues. Further investigations to develop CbA mimetics with more favorable bioactivities and physicochemical properties are ongoing in our laboratory.

## Supporting Information Available

Experimental procedures for peptide synthesis and biological evaluations, characterization of peptides, and supporting figures (PDF)

## ABBREVIATIONS

Alloc: allyloxycarbonyl
ATG5: authophagy-related protein 5
Bph: β-(4-biphenylyl)alanine
DIC: *N*,*N*′-diisopropylcarbodiimide
DIEA: *N*,*N*-diisopropylethylamine
EDCI: 1-ethyl-3-(3-(dimethylamino)propyl)carbodiimide
EGFR: epidermal growth factor receptor
HATU: *O*-(7-aza-1*H*-benzotriazol-1-yl)-*N*,*N*,*N*′,*N*′-tetramethyluronium hexafluorophosphate
HFIP: 1,1,1,3,3,3-hexafluoro-2-propanol
Hva: 2-hydroxyisovaleric acid
HOAt: 1-hydroxy-7-azabenzotriazole
HOBt: 1-hydroxybenzotriazole
Me_2_Val: *N*,*N*-dimethylvaline
MeAla: *N*-methylalanine
MeIle: *N*-methylisoleucine
MeLeu: *N*-methylleucine
MeLys: *N*^α^-methyllysine
MeLys(Me): *N*,*N*′-dimethyllysine
MeOrn: *N*^α^-methylornithine
MeOrn(Me): *N*,*N*′-dimethylornithine
MePhe: *N*-methylphenylalanine
MeSer(Me): *N*,*O*-dimethylserine
MeThr: *N*-methylthreonine
mTOR: mammalian target of rapamycin
Nal: β-naphthylalanine
Ns: *o*-nitrobenzenesulfonyl
Orn: ornithine
Pal: β-pyridylalanine
SPPS: solid-phase peptide synthesis
Tic: 1,2,3,4-tetrahydroisoquinoline-3-carboxylic acid
Tyr(Me): *O*-methyltyrosine
VEGFA: vascular endothelial growth factor A
VEGFR2: vascular endothelial growth factor receptor 2
