# Supplementary material for: Design of Coibamide A Mimetics with Improved Cellular Bioactivity
Source: ACS Med Chem Lett. 2021 Dec 29;13(1):105–10. doi: 10.1021/acsmedchemlett.1c00591 (PMC8762706; doi:10.1021/acsmedchemlett.1c00591)
Supplement: Supplementary file 1 — ml1c00591_si_001.pdf [file ml1c00591_si_001.pdf]

Supporting Information

Design of Coibamide A Mimetics with Improved Cellular Bioactivity

Takashi Kitamura,[†] Rikito Suzuki,[†,§] Shinsuke Inuki,[†] Hiroaki Ohno,[†]
Kerry L. McPhail,[‡] and Shinya Oishi*[,†,§]

[†]*Graduate School of Pharmaceutical Sciences, Kyoto University, Sakyo-ku, Kyoto 606-8501, Japan*

[‡]*Department of Pharmaceutical Sciences, College of Pharmacy, Oregon State University, Corvallis, Oregon 97331, United States*

[§]*Department of Medicinal Chemistry, Kyoto Pharmaceutical University, Yamashina-ku, Kyoto 607-8412, Japan*

E-mail: soishi@mb.kyoto-phu.ac.jp

<u>*Table of Contents*</u>

Experimental section S2
Structures of reported Sec61 inhibitors S13
Synthesis of [Bph10]-coibamide A S14
References S15
NMR spectra S16

Experimental section

Synthetic general method

^{1}H and ^{13}C NMR spectra were recorded using a JEOL ECA-500 spectrometer. Chemical shifts are reported in *δ* (ppm), relative to Me_4Si (in $CDCl_3$) as an internal standard for ^{1}H, and referenced to the residual solvent signal for ^{13}C. Exact mass (HRMS) data were recorded on a Shimadzu LC-ESI-IT-TOF-MS equipment. Optical rotations were measured using a JASCO P-1020 polarimeter. For flash chromatography, Wakogel C-300E (Wako) was employed. For analytical HPLC, a Cosmosil 5C18-ARII column (4.6 × 250 mm, Nacalai Tesque, Inc.) was employed with a linear gradient of CH_3CN (with 0.1% (v/v) TFA) in H_2O, and eluting products were detected by UV at 220 nm. Preparative HPLC was performed using a Cosmosil 5C18-ARII preparative column (20 × 250 mm, Nacalai Tesque, Inc.). The compound purity for the bioassays was determined to be >90% by HPLC analysis.

Fmoc-MeSer(Me)-OH. Fmoc-MeSer(Me)-OH was synthesized by the identical procedure reported previously.[S1,S2] To a suspension of Fmoc-Ser(Me)-OH (5.74 g, 16.8 mmol) in toluene (300 mL), paraformaldehyde (3.33 g, 111 mmol) and $TsOH·H_2O$ (320 mg, 1.68 mmol) were added, and the mixture was refluxed for 2 h. The solution was washed with aqueous $NaHCO_3$ and brine, and dried over $MgSO_4$. After concentration, the crystalline product was dissolved in $CHCl_3$/TFA (1:1, 170 mL), and Et_3SiH (8.04 mL, 50.4 mmol) was added. The solution was stirred at room temperature for 22 h followed by concentration to give an oily residue. Purification by flash chromatography on silica gel ($CHCl_3$:MeOH = 1:0 to 20:1) provided Fmoc-MeSer(Me)-OH as a colorless oil (5.87 g, 98% for 2 steps). The spectral data were in good agreement with those previously reported.[S2]

Alloc-Bph-OH. To a suspension of H-Bph-OH (419 mg, 1.74 mmol) in THF (0.91 mL) and H_2O (1.93 mL), allyl chloroformate (185 μL, 1.74 mmol) and 2.0 M aqueous NaOH (1.74 mL) were added at room temperature. After being stirred for 3.5 h, the reaction mixture was concentrated. The residue was acidified with 2.0 M HCl, and extracted with EtOAc. The extract was washed with brine, and dried over $MgSO_4$. After concentration, the residue was recrystallized from *n*-hexane-EtOAc to provide Alloc-Bph-OH as a white solid (489 mg, 86%): $[\alpha]^{25}_{D}$ +76.2 (*c* 0.52, $CHCl_3$); ^{1}H NMR (500 MHz, $CDCl_3$) δ: 3.12-3.28 (m, 2H), 4.57 (d, *J* = 5.5 Hz, 2H), 4.69-4.76 (m, 1H), 5.14-5.24 (m, 2H), 5.28 (d, *J* = 17.0 Hz, 1H) 5.87-5.93 (m, 1H), 7.25 (d, *J* = 7.5 Hz, 2H), 7.33 (t, *J* = 7.5 Hz, 1H), 7.42 (t, *J* = 7.5 Hz, 2H), 7.48-7.62 (m, 4H); ^{13}C NMR (125 MHz, $CDCl_3$) δ: 37.3, 54.5, 66.0, 118.0, 127.0, 127.3, 127.4, 128.8, 129.7, 132.4, 134.5, 140.1, 140.6, 155.8, 176.1; HRMS (ESI-TOF) calcd for $C_{19}H_{20}NO_4$ $[M+H]^+$: 326.1387; found: 326.1388.

Preparation of N-terminal ester moiety.

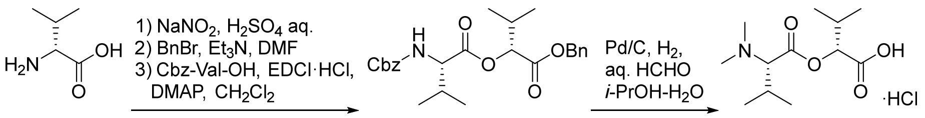

D-Val S1 S2

Cbz-Val-D-Hva-OBn (S1). To a solution of D-valine (27.9 g, 238 mmol) in 0.5 M H_2SO_4 (950 mL), a solution of $NaNO_2$ (98.2 g, 1.43 mol) in H_2O (325 mL) was added dropwise at 0 °C. The mixture was stirred for 10 h at room temperature. The whole was extracted with Et_2O and the extract was washed with brine, and dried over $MgSO_4$. The filtrate was concentrated under reduced pressure to give crude H-D-Hva-OH (25.4 g), which was used for the next step without further purification. To a stirred solution of crude H-D-Hva-OH (20.7 g, ca. 175 mmol) and Et_3N (48.8 mL, 350 mmol) in dry DMF (100 mL), BnBr (19.8 mL, 166 mmol) was added dropwise at 0 °C. The mixture was stirred for 18 h at room temperature. The resulting mixture was diluted with EtOAc and the whole was washed with citric acid aq., $NaHCO_3$ aq., and brine, and dried over $MgSO_4$. The filtrate was concentrated under reduced pressure to give crude H-D-Hva-OBn (18.9 g), which was used for the next step without further purification. To a mixture of H-D-Hva-OBn (18.9 g, ca. 90.6 mmol), Cbz-Val-OH (27.3 g, 109 mmol) and DMAP (1.11 g, 9.06 mmol) in dry CH_2Cl_2 (180 mL), EDCI·HCl (34.6 g, 181 mmol) was added at 0 °C. The mixture was stirred for 24 h at room temperature. After concentration, the residue was diluted with EtOAc. The whole was washed with citric acid aq., $NaHCO_3$ aq., and brine, and dried over $MgSO_4$. Purification by flash column chromatography on silica gel (*n*-hexane:EtOAc = 5:1) provided compound **S1** as a colorless oil (38.0 g, 44% for 3 steps): $[\alpha]^{26}_D$ +15.3 (*c* 0.79, $CHCl_3$); ^1H NMR (500 MHz, $CDCl_3$) δ: 0.89-1.00 (m, 12H), 2.19-2.43 (m, 2H), 4.44 (dd, *J* = 9.5, 4.0 Hz, 1H), 4.90 (d, *J* = 4.0 Hz, 1H), 5.11-5.21 (m, 4H), 5.27 (d, *J* = 9.5 Hz, 1H), 7.24-7.40 (m, 10H); ^{13}C NMR (125 MHz, $CDCl_3$) δ: 17.0, 17.2, 18.7, 19.0, 30.0, 31.1, 59.1, 66.9, 67.0, 77.4, 128.10, 128.13, 128.3, 128.4, 128.46, 128.49, 135.1, 136.1, 156.1, 169.0, 171.4; HRMS (ESI-TOF) calcd for $C_{25}H_{31}NNaO_6$ $[M+Na]^+$: 464.2044; found: 464.2044.

Me_2Val-D-Hva-OH·HCl (S2). To a solution of Cbz-Val-D-Hva-OBn **S1** (304 mg, 0.688 mmol) in *i*-PrOH (2.4 mL) was added 10% Pd/C (42.4 mg) and formaldehyde (37%, 513 μL, 6.88 mmol). The mixture was stirred under H_2 at room temperature for 24 h. The resulting mixture was filtered using a membrane filter (Advantec T050A025A, 0.50 μm) and concentrated under reduced pressure. The residue was purified by a CombiFlash® Rf 150 apparatus using a C18 reversed phase column to provide TFA salt. To replace TFA counterions with chloride ions, the product was lyophilized in the presence of HCl, which gave **S2** as a white solid (133 mg, 68%): $[\alpha]^{25}_D$ +8.59 (*c* 0.55, $CHCl_3$); ^1H NMR (500 MHz, $CDCl_3$) δ: 1.00 (d, *J* = 7.0 Hz, 3H), 1.02-1.09 (m, 6H), 1.31 (d, *J* = 6.5 Hz, 3H), 2.34-2.44 (m, 2H), 3.00 (s, 6H), 3.88 (d, *J* = 7.0 Hz, 1H), 5.02 (d, *J* = 3.5 Hz, 1H); ^{13}C NMR (125

MHz, $CDCl_3$) δ: 16.7, 17.8, 18.9, 20.0, 27.5, 29.2, 41.6, 71.9, 78.8, 167.0, 169.8; HRMS (ESI-TOF) calcd for $C_{12}H_{24}NO_4$ $[M+H]^+$: 246.1700; found: 246.1699.

General procedure for solid-phase peptide synthesis.

Loading of an amino acid on the solid support. A solution of Fmoc amino acid (0.198 mmol) and DIEA (138 μL, 0.792 mmol) in dry CH_2Cl_2 (2.0 mL), was added to (2-Cl)Trt chloride resin (204 mg, 0.326 mmol). The reaction was continued for 2 h at room temperature.

Deprotection of Fmoc group. The Fmoc-protected peptidyl resin was treated with 20% piperidine/DMF for 20 min.

Coupling reaction using HATU/DIEA. DIEA (104 μL, 0.600 mmol) was added to a solution of Fmoc amino acid (0.30 mmol) and HATU (110 mg, 0.290 mmol) in DMF. The whole was poured into the peptidyl resin (0.10 mmol), and the reaction was continued for 1.5 h at 40 °C. For the coupling of N-terminal ester moiety (**S2**), an excess amount of DIEA (157 μL, 0.900 mmol) was used.

Coupling reaction using DIC/HOBt. DIC (46 μL, 0.30 mmol) was added to a solution of Fmoc amino acid (0.30 mmol) and HOBt·H_2O (46 mg, 0.30 mmol) in DMF. The whole was poured into the peptidyl resin (0.100 mmol), and the reaction was continued for 1.5 h at 40 °C.

N-Methylation on solid support.[S4] 2,4,6-Collidine (132 μL, 1.00 mmol) was added to a solution of NsCl (111 mg, 0.500 mmol) in NMP. The whole was poured into the peptidyl resin (0.100 mmol), and the reaction was continued for 15 min at room temperature. After removal of the reagent solution, a solution of MeOH (20 μL, 0.50 mmol) and Ph_3P (131 mg, 0.500 mmol) in dry THF was added into the peptidyl resin. DEAD (228 μL, 0.500 mmol) was added dropwise, and the reaction was continued for 30 min at room temperature. This reaction was repeated twice. To a suspension of the peptidyl resin in NMP, DBU (75 μL, 0.50 mmol) and 2-mercaptoethanol (70 μL, 1.0 mmol) were added, and the reaction was continued for 5 min. This deprotection process was repeated twice.

Deprotection of Alloc group. To the peptidyl resin (0.100 mmol) were added $PhSiH_3$ (247 μL, 2.00 mmol) and $Pd(PPh_3)_4$ (23 mg, 0.020 mmol) in dry CH_2Cl_2, and the reaction was continued for 10 min.

Cleavage from the resin. The peptidyl resin was treated with 1,1,1,3,3,3-hexafluoropropan-2-ol (HFIP)/CH_2Cl_2 (3:7) for 2 h at room temperature. After filtration of the residual resin, the filtrate was concentrated under reduced pressure to give a crude peptide, which was used for the next step without further purification.

[MeAla^3, MeLys(Me)^5, MeAla^6]-Coibamide A (2a). The linear peptide was constructed by solid-phase peptide synthesis on peptidyl resin **3** (0.490 mmol/g, 204 mg, 0.100 mmol). After the cleavage from the resin **7** as described above, EDCI·HCl (192 mg, 1.00 mmol) was added to a solution of linear

peptide, HOAt (136 mg, 1.00 mmol), and DIEA (697 μL, 4.00 mmol) in dry DMF (100 mL) at 0 °C. The reaction mixture was allowed to warm up to room temperature and the stirring was continued for 18 h. The reaction mixture was concentrated and the residue was purified by RP-HPLC to give **2a** (5.9 mg, 4.4% from resin) as a white powder: ^{1}H NMR (500 MHz, $CDCl_3$, mixture of rotamers) δ: 0.69-1.04 (m, 27H), 1.08-1.15 (m, 4H), 1.15-1.22 (m, 4H), 1.22-1.29 (m, 4H), 1.29-1.37 (m, 4H), 1.37-1.76 (m, 9H), 1.76-1.97 (m, 2H), 2.00-2.29 (m, 3H), 2.42-2.53 (m, 1H), 2.57-2.74 (m, 3H), 2.74-3.15 (m, 27H), 3.78 (s, 2H), 3.79 (s, 1H), 3.83-3.90 (m, 1H), 4.23 (d, J = 11.0 Hz, 1H), 4.29-4.41 (m, 0.5H), 4.85-5.17 (m, 3.5H), 5.35-5.57 (m, 4H), 6.47 (d, J = 8.5 Hz, 0.5H), 6.74-6.87 (m, 2.5H), 6.99 (d, J = 8.5 Hz, 0.5H), 7.07 (d, J = 8.5 Hz, 1H), 7.08-7.18 (m, 0.5H), 8.02 (br s, 0.5H), 8.28 (d, J = 10.0 Hz, 0.5H); ^{13}C NMR (125 MHz, $CDCl_3$, mixture of rotamers) δ: 9.9, 11.3, 14.1, 14.37, 14.44, 15.3, 15.5, 15.6, 16.3, 16.4, 18.0, 18.3, 18.69, 18.74, 18.8, 19.0, 20.0, 20.8, 21.2, 21.3, 21.57, 21.62, 22.2, 22.6, 23.0, 23.2, 24.4, 24.65, 24.74, 24.9, 25.3, 26.1, 26.4, 27.78, 27.81, 28.6, 28.7, 28.8, 28.9, 29.0, 29.3, 29.55, 29.60, 29.7, 29.9, 30.1, 32.2, 32.5, 33.0, 33.4, 35.2, 37.3, 37.4, 37.5, 37.7, 38.9, 39.4, 42.1, 43.9, 46.3, 48.3, 48.6, 49.7, 49.8, 50.2, 50.5, 50.8, 51.1, 51.3, 52.3, 52.9, 54.9, 58.4, 65.2, 70.6, 70.6, 113.4, 113.8, 127.7, 127.8, 129.9, 130.2, 158.3, 158.4, 167.3, 167.4, 167.6, 168.2, 168.6, 169.4, 170.2, 170.3, 170.4, 170.5, 170.9, 171.0, 171.1, 172.1, 172.4, 172.4; HRMS (ESI-TOF) calcd for $C_{62}H_{107}N_{10}O_{12}$ $[M+H]^+$: 1183.8064; found: 1183.8070.

[MeAla³, D-MeLys(Me)⁵, MeAla⁶]-Coibamide A (2b). According to the procedure described for the preparation of **2a**, peptidyl resin **3** (0.690 mmol/g, 145 mg, 0.100 mmol) was converted into **2b** (10.3 mg, 8% from resin) as a white powder: HRMS (ESI-TOF) calcd for $C_{62}H_{106}N_{10}NaO_{12}$ $[M+Na]^+$:1205.7884; found: 1205.7864.

[MeAla³, MeOrn(Me)⁵, MeAla⁶]-Coibamide A (2c). According to the procedure described for the preparation of **2a**, peptidyl resin **3** (0.690 mmol/g, 145 mg, 0.100 mmol) was converted into **2c** (27.6 mg, 22% from resin) as a white powder: HRMS (ESI-TOF) calcd for $C_{61}H_{105}N_{10}O_{12}$ $[M+H]^+$: 1169.7908; found: 1169.7909.

[MeAla³, D-MeOrn(Me)⁵, MeAla⁶]-Coibamide A (2d). According to the procedure described for the preparation of **2a**, peptidyl resin **3** (0.690 mmol/g, 145 mg, 0.100 mmol) was converted into **2d** (9.5 mg, 7% from resin) as a white powder: HRMS (ESI-TOF) calcd for $C_{61}H_{104}N_{10}NaO_{12}$ $[M+Na]^+$: 1191.7727; found: 1191.7740.

[MeAla³, MeLys⁵, MeAla⁶]-Coibamide A (2e). According to the procedure described for the

preparation of **2a**, peptidyl resin **3** (0.490 mmol/g, 204 mg, 0.100 mmol) was converted into **2e** (25.6 mg, 20% from resin) as a white powder: HRMS (ESI-TOF) calcd for $C_{61}H_{105}N_{10}O_{12}$ $[M+H]^+$: 1169.7908; found: 1169.7909.

[MeAla^3, D-MeLys^5, MeAla^6]-Coibamide A (2f). According to the procedure described for the preparation of **2a**, peptidyl resin **3** (0.690 mmol/g, 145 mg, 0.100 mmol) was converted into **2f** (17.2 mg, 13% from resin) as a white powder: HRMS (ESI-TOF) calcd for $C_{61}H_{105}N_{10}O_{12}$ $[M+H]^+$: 1169.7908; found: 1169.7906.

[MeAla^3, MeOrn^5, MeAla^6]-Coibamide A (2g). According to the procedure described for the preparation of **2a**, peptidyl resin **3** (0.690 mmol/g, 145 mg, 0.100 mmol) was converted into **2g** (19.4 mg, 15% from resin) as a white powder: HRMS (ESI-TOF) calcd for $C_{60}H_{103}N_{10}O_{12}$ $[M+H]^+$: 1155.7751; found: 1155.7756.

[MeAla^3, D-MeOrn ^{5}, MeAla^6]-Coibamide A (2h). According to the procedure described for the preparation of **2a**, peptidyl resin **3** (0.690 mmol/g, 145 mg, 0.100 mmol) was converted into **2h** (19.8 mg, 16% from resin) as a white powder: HRMS (ESI-TOF) calcd for $C_{60}H_{103}N_{10}O_{12}$ $[M+H]^+$: 1155.7751; found: 1155.7753.

[Ala^3, MeLys(Me)^5, MeAla^6]-Coibamide A (8a). According to the procedure described for the preparation of **2a**, peptidyl resin **3** (0.690 mmol/g, 145 mg, 0.100 mmol) was converted into **8a** (6.9 mg, 5% from resin) as a white powder: HRMS (ESI-TOF) calcd for $C_{61}H_{104}N_{10}NaO_{12}$ $[M+Na]^+$: 1191.7727; found: 1191.7742.

[MeAla^3, Leu^4, MeLys(Me)^5, MeAla^6]-Coibamide A (8b). According to the procedure described for the preparation of **2a**, peptidyl resin **3** (0.690 mmol/g, 145 mg, 0.100 mmol) was converted into **8b** (5.4 mg, 4% from resin) as a white powder: HRMS (ESI-TOF) calcd for $C_{61}H_{106}N_{10}O_{12}$ $[M+2H]^{2+}$: 585.3991; found: 585.3975.

[MeAla^3, Lys(Me)^5, MeAla^6]-Coibamide A (8c). According to the procedure described for the preparation of **2a**, peptidyl resin **3** (0.690 mmol/g, 145 mg, 0.100 mmol) was converted into **8c** (8.5 mg, 7% from resin) as a white powder: HRMS (ESI-TOF) calcd for $C_{61}H_{105}N_{10}O_{12}$ $[M+H]^+$: 1169.7908; found: 1169.7922.

[MeAla[3], MeLys(Me)[5], Ala[6]]-Coibamide A (8d). According to the procedure described for the preparation of **2a**, peptidyl resin **3** (0.690 mmol/g, 145 mg, 0.100 mmol) was converted into **8d** (8.8 mg, 7% from resin) as a white powder: HRMS (ESI-TOF) calcd for $C_{61}H_{106}N_{10}O_{12}$ $[M+2H]^{2+}$: 585.3991; found: 585.3976.

[MeAla[3], MeLys(Me)[5], MeAla[6], Ile[7]]-Coibamide A (8e). According to the procedure described for the preparation of **2a**, peptidyl resin **3** (0.690 mmol/g, 145 mg, 0.100 mmol) was converted into **8e** (11.6 mg, 9% from resin) as a white powder: HRMS (ESI-TOF) calcd for $C_{61}H_{106}N_{10}O_{12}$ $[M+2H]^{2+}$: 585.3991; found: 585.3990.

[MeAla[3], MeLys(Me)[5], MeAla[6], Leu[9]]-Coibamide A (8f). According to the procedure described for the preparation of **2a**, peptidyl resin **3** (0.690 mmol/g, 145 mg, 0.100 mmol) was converted into **8f** (11.6 mg, 9% from resin) as a white powder: HRMS (ESI-TOF) calcd for $C_{61}H_{104}N_{10}NaO_{12}$ $[M+Na]^{+}$: 1191.7727; found: 1191.7734.

[MeAla[3], MeLys(Me)[5], D-MeAla[6]]-Coibamide A (9a). According to the procedure described for the preparation of **2a**, peptidyl resin **3** (0.690 mmol/g, 145 mg, 0.100 mmol) was converted into **9a** (3.5 mg, 3% from resin) as a white powder: HRMS (ESI-TOF) calcd for $C_{62}H_{107}N_{10}O_{12}$ $[M+H]^{+}$: 1183.8064; found: 1183.8070.

[MeAla[3], MeLys(Me)[5], MeAla[6], D-*allo*-MeIle[7]]-Coibamide A (9b). According to the procedure described for the preparation of **2a**, peptidyl resin **3** (0.690 mmol/g, 145 mg, 0.100 mmol) was converted into **9b** (2.3 mg, 2% from resin) as a white powder: HRMS (ESI-TOF) calcd for $C_{62}H_{107}N_{10}O_{12}$ $[M+H]^{+}$: 1183.8064; found: 1183.8066.

[MeAla[3], MeLys(Me)[5], MeAla[6], D-Ala[8]]-Coibamide A (9c). According to the procedure described for the preparation of **2a**, peptidyl resin **3** (0.690 mmol/g, 145 mg, 0.100 mmol) was converted into **9c** (3.2 mg, 3% from resin) as a white powder: HRMS (ESI-TOF) calcd for $C_{62}H_{106}N_{10}NaO_{12}$ $[M+Na]^{+}$:1205.7884; found: 1205.7830.

[MeAla[3], MeLys(Me)[5], MeAla[6], D-MeLeu[9]]-Coibamide A (9d). According to the procedure described for the preparation of **2a**, peptidyl resin **3** (0.570 mmol/g, 175 mg, 0.100 mmol) was converted into **9d** (13.4 mg, 10% from resin) as a white powder: HRMS (ESI-TOF) calcd for $C_{62}H_{107}N_{10}O_{12}$ $[M+H]^{+}$: 1183.8064; found: 1183.8063.

[MeAla[3], MeLys(Me)[5], MeAla[6], D-Tyr(Me)[10]]-Coibamide A (9e). According to the procedure described for the preparation of **2a**, peptidyl resin **3** (0.490 mmol/g, 204 mg, 0.100 mmol) was converted into **9e** (4.4 mg, 3% from resin) as a white powder: HRMS (ESI-TOF) calcd for $C_{62}H_{107}N_{10}O_{12}$ $[M+H]^+$: 1183.8064; found: 1183.8059.

[MeAla[3], MeLys(Me)[5], MeAla[6], Bph[10]]-Coibamide A (10j). According to the procedure described for the preparation of **2a**, peptidyl resin **3** (0.490 mmol/g, 204 mg, 0.100 mmol) was converted into **10j** (16.2 mg, 12% from resin) as a white powder: ^{1}H NMR (500 MHz, $CDCl_3$, mixture of rotamers) δ: 0.66-1.79 (m, 51H), 1.79-2.00 (m, 2H), 2.00-2.32 (m, 3H), 2.41-2.85 (m, 9H), 2.85-3.31 (m, 22H), 3.78-3.95 (m, 1H), 4.21 (m, 0.5H), 4.28-4.46 (m, 0.5H), 4.74-5.21 (m, 4H), 5.21-5.69 (m, 4H), 6.52 (d, J = 8.5 Hz, 0.5H), 6.83 (br s, 0.5H), 7.18 (d, J = 8.0 Hz, 1H), 7.24 (d, J = 8.0 Hz, 1H), 7.31-7.39 (m, 1H), 7.39-7.48 (m, 2H), 7.52 (d, J = 8.0 Hz, 1H), 7.55-7.69 (m, 3H), 7.97 (br s, 0.5H), 8.52 (d, J = 9.5 Hz, 0.5H); ^{13}C NMR (125 MHz, $CDCl_3$, mixture of rotamers) δ: 10.2, 11.7, 14.5, 14.7, 14.8, 15.5, 15.8, 16.0, 16.7, 16.9, 18.3, 18.6, 19.1, 19.18, 19.22, 19.3, 20.4, 21.2, 21.6, 21.9, 22.0, 22.7, 23.0, 23.3, 23.6, 24.8, 25.0, 25.1, 25.2, 25.4, 26.6, 26.9, 28.16, 28.20, 29.00, 29.04, 29.1, 29.3, 29.7, 29.91, 29.94, 30.1, 30.2, 30.3, 30.4, 32.5, 32.8, 33.5, 33.8, 35.6, 37.7, 37.8, 37.9, 38.5, 39.5, 39.9, 42.5, 44.4, 46.6, 48.8, 49.0, 50.0, 50.1, 50.4, 50.6, 51.5, 51.6, 52.6, 53.4, 58.8, 65.5, 70.8, 70.9, 126.8, 126.9, 127.0, 127.3, 127.4, 127.5, 128.8, 128.9, 129.7, 130.3, 135.1, 135.4, 139.7, 139.9, 140.1, 140.5, 167.7, 167.8, 167.9, 168.5, 169.1, 169.9, 170.6, 170.8, 170.9, 171.25, 171.29, 171.34, 172.5, 172.6, 172.7; HRMS (ESI-TOF) calcd for $C_{67}H_{109}N_{10}O_{11}$ $[M+H]^+$: 1229.8272; found: 1229.8254.

[MeAla[3], MeLys(Me)[5], MeAla[6], Phe[10]]-Coibamide A (10a). According to the procedure described for the preparation of **2a**, peptidyl resin **6** (0.100 mmol) was converted into **10a** (4.2 mg, 3% from resin) as a white powder: HRMS (ESI-TOF) calcd for $C_{61}H_{105}N_{10}O_{11}$ $[M+H]^+$: 1153.7959; found: 1153.7958.

[MeAla[3], MeLys(Me)[5], MeAla[6], Phe(4-NO_2)[10]]-Coibamide A (10b). According to the procedure described for the preparation of **2a**, peptidyl resin **6** (0.100 mmol) was converted into **10b** (17.4 mg, 13% from resin) as a white powder: HRMS (ESI-TOF) calcd for $C_{61}H_{104}N_{11}O_{13}$ $[M+H]^+$: 1198.7810; found: 1198.7809.

[MeAla[3], MeLys(Me)[5], MeAla[6], Phe(4-CF_3)[10]]-Coibamide A (10c). According to the procedure described for the preparation of **2a**, peptidyl resin **6** (0.100 mmol) was converted into **10c** (32.8 mg,

25% from resin) as a white powder: HRMS (ESI-TOF) calcd for $C_{62}H_{104}F_3N_{10}O_{11}$ $[M+H]^+$: 1221.7833; found: 1221.7832.

[MeAla^3, MeLys(Me)^5, MeAla^6, Phe(4-CN)^{10}]-Coibamide A (10d). According to the procedure described for the preparation of **2a**, peptidyl resin **6** (0.100 mmol) was converted into **10d** (23.9 mg, 18% from resin) as a white powder: HRMS (ESI-TOF) calcd for $C_{62}H_{104}N_{11}O_{11}$ $[M+H]^+$: 1178.7911; found: 1178.7911.

[MeAla^3, MeLys(Me)^5, MeAla^6, Phe(4-N$_3$)^{10}]-Coibamide A (10e). According to the procedure described for the preparation of **2a**, peptidyl resin **6** (0.100 mmol) was converted into **10e** (5.8 mg, 4% from resin) as a white powder: HRMS (ESI-TOF) calcd for $C_{61}H_{104}N_{13}O_{11}$ $[M+H]^+$: 1194.7973; found: 1194.7974.

[MeAla^3, MeLys(Me)^5, MeAla^6, Phe(4-Cl)^{10}]-Coibamide A (10f). According to the procedure described for the preparation of **2a**, peptidyl resin **6** (0.100 mmol) was converted into **10f** (25.9 mg, 20% from resin) as a white powder: HRMS (ESI-TOF) calcd for $C_{61}H_{104}ClN_{10}O_{11}$ $[M+H]^+$: 1187.7569; found: 1187.7564.

[MeAla^3, MeLys(Me)^5, MeAla^6, Phe(4-*t*-Bu)^{10}]-Coibamide A (10g). According to the procedure described for the preparation of **2a**, peptidyl resin **6** (0.100 mmol) was converted into **10g** (36.1 mg, 27% from resin) as a white powder: HRMS (ESI-TOF) calcd for $C_{65}H_{113}N_{10}O_{11}$ $[M+H]^+$: 1209.8585; found: 1209.8582.

[MeAla^3, MeLys(Me)^5, MeAla^6, Phe(4-O*t*-Bu)^{10}]-Coibamide A (10h). According to the procedure described for the preparation of **2a**, peptidyl resin **6** (0.100 mmol) was converted into **10h** (31.7 mg, 24% from resin) as a white powder: HRMS (ESI-TOF) calcd for $C_{65}H_{113}N_{10}O_{12}$ $[M+H]^+$: 1225.8534; found: 1225.8534.

[MeAla^3, MeLys(Me)^5, MeAla^6, Phe(4-OCF$_3$)^{10}]-Coibamide A (10i). According to the procedure described for the preparation of **2a**, peptidyl resin **6** (0.100 mmol) was converted into **10i** (21.8 mg, 16% from resin) as a white powder: HRMS (ESI-TOF) calcd for $C_{62}H_{104}F_3N_{10}O_{12}$ $[M+H]^+$: 1237.7782; found: 1237.7784.

[MeAla^3, MeLys(Me)^5, MeAla^6, 2-Pal^{10}]-Coibamide A (10k). According to the procedure

described for the preparation of **2a**, peptidyl resin **6** (0.100 mmol) was converted into **10k** (7.1 mg, 5% from resin) as a white powder: HRMS (ESI-TOF) calcd for $C_{60}H_{105}N_{11}O_{11}$ $[M+2H]^{2+}$: 577.8992; found: 577.8993.

[MeAla^{3}, MeLys(Me)^{5}, MeAla^{6}, 3-Pal^{10}]-Coibamide A (10l). According to the procedure described for the preparation of **2a**, peptidyl resin **6** (0.100 mmol) was converted into **10l** (31.3 mg, 23% from resin) as a white powder: HRMS (ESI-TOF) calcd for $C_{60}H_{105}N_{11}O_{11}$ $[M+2H]^{2+}$: 577.8992; found: 577.8992.

[MeAla^{3}, MeLys(Me)^{5}, MeAla^{6}, 4-Pal^{10}]-Coibamide A (10m). According to the procedure described for the preparation of **2a**, peptidyl resin **6** (0.100 mmol) was converted into **10m** (25.0 mg, 18% from resin) as a white powder: HRMS (ESI-TOF) calcd for $C_{60}H_{105}N_{11}O_{11}$ $[M+2H]^{2+}$: 577.8992; found: 577.8991.

[MeAla^{3}, MeLys(Me)^{5}, MeAla^{6}, MePhe^{10}]-Coibamide A (10n). According to the procedure described for the preparation of **2a**, peptidyl resin **6** (0.100 mmol) was converted into **10n** (2.0 mg, 2% from resin) as a white powder: HRMS (ESI-TOF) calcd for $C_{62}H_{107}N_{10}O_{11}$ $[M+H]^{+}$: 1167.8115; found: 1167.8116.

[MeAla^{3}, MeLys(Me)^{5}, MeAla^{6}, Tic^{10}]-Coibamide A (10o). According to the procedure described for the preparation of **2a**, peptidyl resin **6** (0.100 mmol) was converted into **10o** (8.9 mg, 7% from resin) as a white powder: HRMS (ESI-TOF) calcd for $C_{62}H_{105}N_{10}O_{11}$ $[M+H]^{+}$: 1165.7959; found: 1165.7959.

[MeAla^{3}, MeLys(Me)^{5}, MeAla^{6}, 1-Nal^{10}]-Coibamide A (10p). According to the procedure described for the preparation of **2a**, peptidyl resin **6** (0.100 mmol) was converted into **10p** (5.8 mg, 4% from resin) as a white powder: HRMS (ESI-TOF) calcd for $C_{65}H_{107}N_{10}O_{11}$ $[M+H]^{+}$: 1203.8115; found: 1203.8114.

[MeAla^{3}, MeLys(Me)^{5}, MeAla^{6}, 2-Nal^{10}]-Coibamide A (10q). According to the procedure described for the preparation of **2a**, peptidyl resin **6** (0.100 mmol) was converted into **10q** (5.5 mg, 4% from resin) as a white powder: HRMS (ESI-TOF) calcd for $C_{65}H_{107}N_{10}O_{11}$ $[M+H]^{+}$: 1203.8115; found: 1203.8112.

[Bph^{10}]-Coibamide A (11). The linear peptides were constructed by solid-phase peptide synthesis on

peptidyl resin **S3** (0.712 mmol/g, 422 mg, 0.300 mmol). Fmoc- or Alloc- protected amino acids (0.900 mmol) were coupled at 40 °C by using DIC (139 μL, 0.900 mmol) and HOAt (122 mg, 0.900 mmol) in DMF. Fmoc-D-MeAla-OH (488 mg, 1.50 mmol) was coupled onto the hydroxy group of MeThr[5] at room temperature using DIC (232 μL, 1.50 mmol) and DMAP (55 mg, 0.45 mmol) in dry DCE. After cleavage from the resin **S7** as described above, HATU (570 mg, 1.50 mmol) and DIEA (1.05 mL, 6.00 mmol) were added to a solution of crude linear peptide in dry CH_2Cl_2 (300 mL). The reaction mixture was stirred for 12 h. After the mixture was concentrated, the residue was purified by RP-HPLC to give **11** (26.5 mg, 6.1% from resin) as a white powder: ^{1}H NMR (500 MHz, $CDCl_3$, mixture of rotamers) δ: 0.61-1.17 (m, 33H), 1.17-1.48 (m, 11H), 1.48-1.61 (m, 2H), 1.61-1.73 (m, 1H), 2.01-2.15 (m, 1H), 2.15-2.33 (m, 2H), 2.35-2.87 (m, 9H), 2.87-3.06 (m, 15H), 3.06-3.43 (m, 12H), 3.44-3.57 (m, 1H), 3.57-3.73 (m, 2H), 3.73-3.82 (m, 1H), 3.82-3.95 (m, 2H), 4.71-4.87 (m, 1H), 5.06 (d, J = 4.5 Hz, 1H), 5.13-5.23 (m, 1H), 5.23-5.30 (m, 1H), 5.30-5.46 (m, 1H), 5.46-5.59 (m, 1H), 5.68-5.84 (m, 1H), 5.84-6.05 (m, 1H), 6.20-6.44 (m, 1H), 6.54-6.70 (m, 1H), 6.82-7.11 (m, 1H), 7.25 (d, J = 7.0 Hz, 1H), 7.29-7.39 (m, 2H), 7.39-7.60 (m, 6H); ^{13}C NMR (125 MHz, $CDCl_3$, mixture of rotamers) δ: 11.6, 12.9, 13.0, 15.8, 16.6, 17.5, 18.6, 18.7, 19.0, 19.1, 21.0, 21.11, 21.14, 21.5, 22.1, 23.1, 23.2, 23.3, 23.4, 24.3, 25.3, 28.2, 28.9, 29.1, 29.7, 29.9, 30.1, 30.2, 30.4, 31.0, 32.0, 36.6, 37.6, 38.8, 39.7, 47.0, 50.0, 51.3, 51.5, 51.7, 52.9, 53.1, 53.3, 58.8, 63.6, 64.7, 68.2, 68.6, 68.9, 69.0, 71.0, 77.6, 126.7, 126.88, 126.93, 127.1, 127.2, 127.4, 128.8, 129.7, 130.0, 130.1, 135.3, 140.0, 140.5, 167.2, 168.0, 168.5, 168.6, 169.15, 169.22, 169.6, 170.0, 170.3, 170.6, 170.7, 171.2, 171.6, 172.0; HRMS (ESI-TOF) calcd for $C_{70}H_{114}N_{10}O_{15}$ $[M+2H]^{2+}$: 667.4227; found: 667.4227.

[MeAla[3], MeAla[6], Bph[10]]-Coibamide A (12). According to the procedure described for the preparation of **11**, peptidyl resin **S3** (0.712 mmol/g, 422 mg, 0.300 mmol) was converted into **12** (30.3 mg, 7.3% from resin) as a white powder: ^{1}H NMR (500 MHz, $CDCl_3$, mixture of rotamers) δ: 0.62-0.84 (d, J = 6.0 Hz, 3H), 0.86-0.99 (m, 15H), 0.99-1.06 (m, 8H), 1.08 (d, J = 7.0 Hz, 3H), 1.11 (d, J = 7.0 Hz, 3H), 1.13-1.24 (m, 8H), 1.24-1.34 (m, 9H), 1.34-1.45 (m, 2H), 1.45-1.59 (m, 2H), 1.64-1.74 (m, 1H), 2.01-2.10 (m, 1H), 2.15-2.30 (m, 2H), 2.33-2.72 (m, 4H), 2.76 (s, 3H), 2.87 (s, 3H), 2.89-3.23 (m, 20H), 3.63-3.83 (m, 2H), 3.87 (d, J = 9.0 Hz, 1H), 4.74-4.90 (m, 1H), 5.01 (d, J = 4.5 Hz, 1H), 5.06-5.71 (m, 6H), 6.32-6.61 (m, 1H), 7.23-7.31 (m, 2H), 7.35 (t, J = 7.5 Hz, 1H), 7.44 (t, J = 7.5 Hz, 2H), 7.46-7.60 (m, 4H); ^{13}C NMR (125 MHz, $CDCl_3$, mixture of rotamers) δ: 11.5, 11.7, 12.7, 12.8, 14.0, 14.4, 14.6, 15.6, 15.8, 16.0, 16.7, 18.5, 18.9, 19.0, 21.0, 21.2, 21.9, 23.0, 23.2, 23.4, 24.1, 25.1, 25.2, 28.0, 28.9, 29.0, 29.5, 29.6, 29.9, 30.1, 30.7, 32.1, 36.6, 37.5, 38.0, 38.7, 39.5, 46.4, 46.7, 50.0, 50.2, 50.3, 50.6, 51.1, 51.2, 51.4, 51.6, 57.3, 64.6, 68.8, 70.8, 70.9, 77.1, 126.6, 126.7, 126.8, 127.0, 127.1, 127.3, 128.7, 129.6, 130.0, 134.9, 137.1, 139.1, 140.0, 140.3, 140.6, 167.4, 167.6,

167.7, 168.5, 169.2, 169.9, 170.4, 170.6, 171.4, 171.5, 171.8, 171.9, 172.8, 173.7; HRMS (ESI-TOF) calcd for $C_{68}H_{108}N_{10}NaO_{13}$ $[M+Na]^+$: 1295.7990; found: 1295.7976.

Growth Inhibition Assay[S3]

A549 cells were cultured in Dulbecco's modified Eagle's medium (DMEM; Sigma) supplemented with 10% (v/v) fetal bovine serum at 37 °C in a 5% CO_2-incubator. Growth inhibition assays using A549 cells were performed in 96-well plates (BD Falcon). A549 cells were seeded at 1000 cells/well in 90 μL of culture media, respectively, and were cultured for 24 h. Chemical compounds in DMSO were diluted 50-fold with the culture medium in advance. 30 μL of the chemical diluents were added. The final volume of DMSO in the medium was equal to 0.5% (v/v). The cells under chemical treatment were incubated for a further 72 h. The wells in the plates were washed twice with the cultured medium without phenol-red. After 1-hour incubation with 100 μL of the medium, the cell culture in each well was supplemented with 20 μL of the MTS reagent (Promega), followed by incubation for additional 40 min. Absorbance at 490 nm of each well was measured using a Wallac 1420 ARVO SX multilabel counter (Perkin Elmer). Three experiments were performed per condition and the averages of inhibition rates in each condition were evaluated to determine IC_{50} values using the GraphPad Prism software.

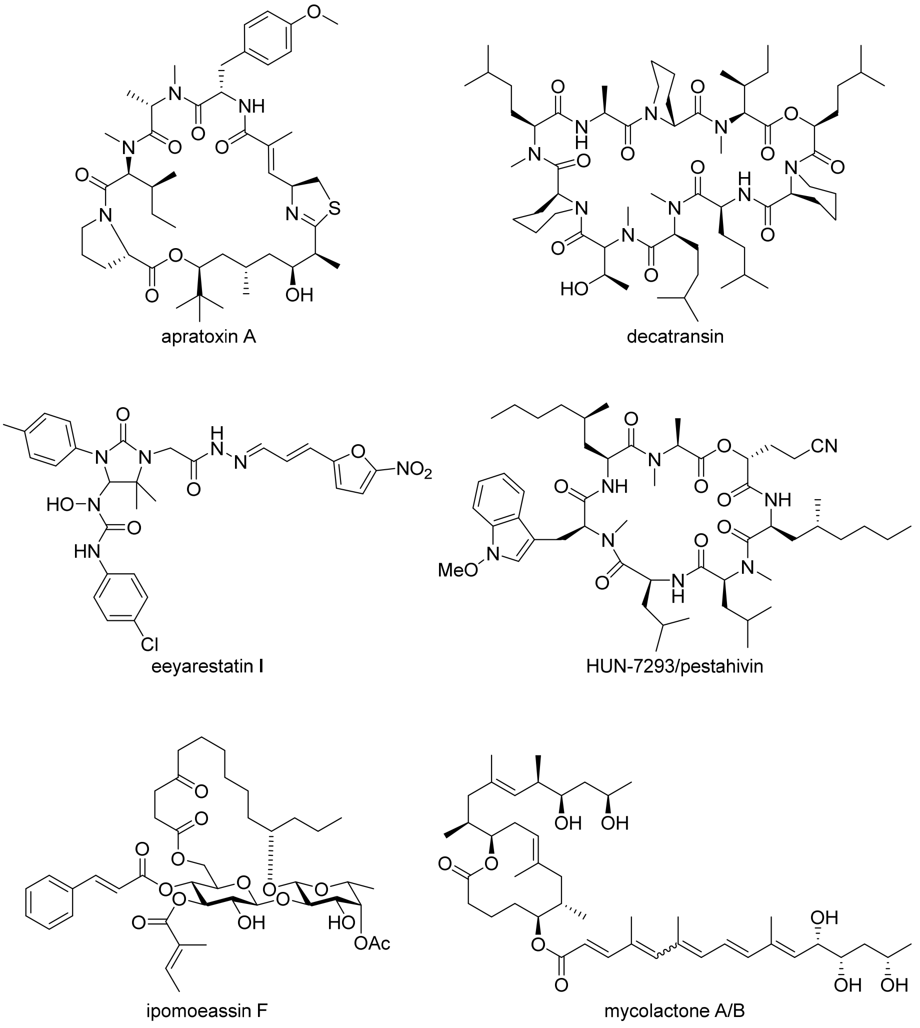

Figure S1. Structures of natural product Sec61 inhibitors.

Synthesis of [Bph^{10}]-Coibamide A

The peptide was assembled on H-MeIle-(2-Cl)Trt resin by Fmoc-SPPS. Coupling of Ser(Me)^{6} followed by on-resin N-methylation[S4] provided peptidyl resin **S5**. MeThr^{5}, MeLeu^{4}, and MeSer(Me)^{3} were efficiently coupled onto *N*-methylamino acids using DIC/HOAt. Coupling of the N-terminal Me_2Val^{1}-D-Hva^{2} moiety using HATU/DIEA gave the peptidyl resin **S6**. Subsequently, Fmoc-D-MeAla-OH was coupled onto the MeThr^{5} hydroxy group using DIC/DMAP. To avoid the diketopiperazine formation under basic condition for deprotection of the Fmoc group, Alloc protection was employed for Bph^{10}, which can be removed under neutral condition. After coupling of Alloc-Bph-OH using DIC/HOAt followed by deprotection of the Alloc group, MeLeu^{9} and Ala^{8} were coupled using DIC/HOBt and DIC/HOAt, respectively. Cleavage of the open-chain peptide **S7** from the resin and macrocyclization with HATU/DIEA in CH_2Cl_2[S5] gave the desired product **11**.

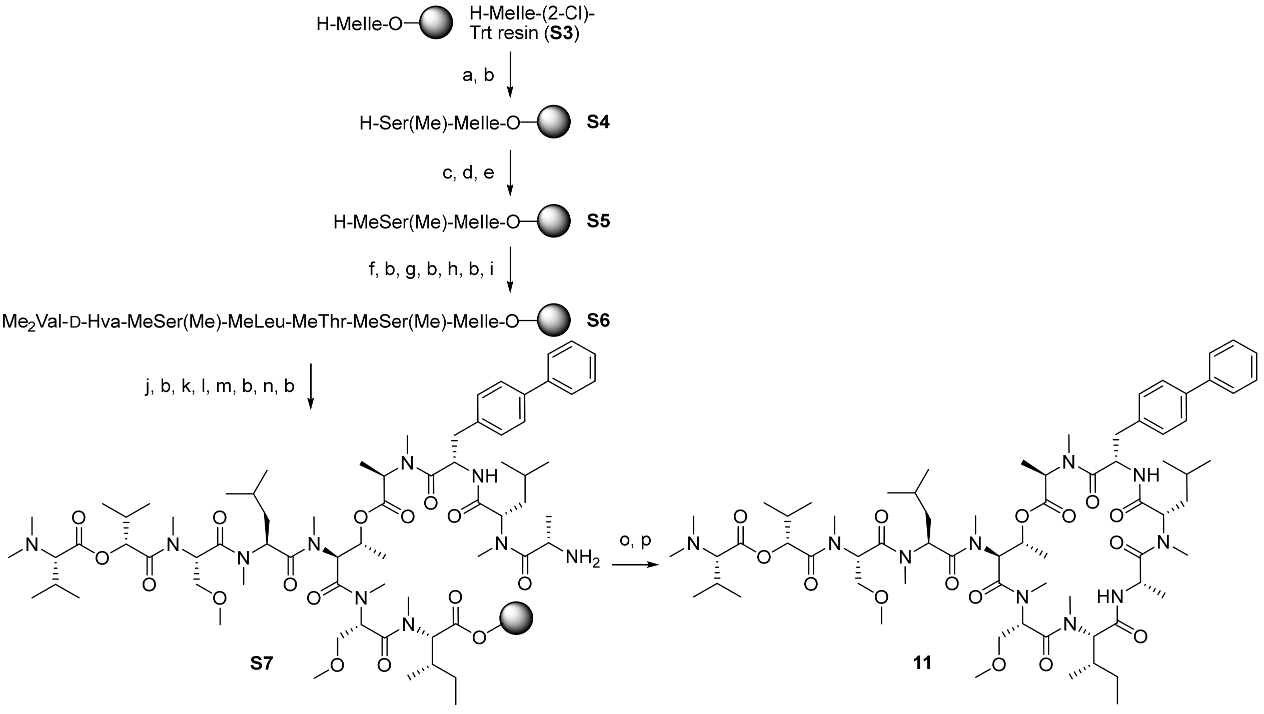

Scheme S1. Synthesis of [Bph^{10}]-coibamide A (**11**). *Reagents and conditions*: (a) Fmoc-Ser(Me)-OH, DIC, HOAt, DMF, 40 °C; (b) 20% piperidine/DMF, rt; (c) NsCl, 2,4,6-collidine, NMP, rt; (d) Ph_3P, DEAD, MeOH, THF, rt; (e) 2-mercaptoethanol, DBU, NMP, rt; (f) Fmoc-MeThr-OH, DIC, HOAt, DMF, 40 °C; (g) Fmoc-MeLeu-OH, DIC, HOAt, DMF, 40 °C (h) Fmoc-MeSer(Me)-OH, DIC, HOAt, DMF, 40 °C; (i) Me_2Val-D-Hva-OH, HATU, DIEA, DMF, 40 °C; (j) Fmoc-D-MeAla-OH, DIC, DMAP, 1,2-DCE, rt; (k) Alloc-Bph-OH, DIC, HOAt, DMF, 40 °C; (l) $Pd(PPh_3)_4$, $PhSiH_3$, CH_2Cl_2, rt; (m) Fmoc-MeLeu-OH, DIC, HOBt·H_2O, DMF, 40 °C; (n) Fmoc-Ala-OH·H_2O, DIC, HOAt, DMF, 40 °C; (o) 30% HFIP/CH_2Cl_2, rt; (p) HATU, DIEA, CH_2Cl_2.

References and Notes

S1 Freidinger, R. M.; Hinkle, J. S.; Perlow, D. S.; Arison, B. H. Synthesis of 9-fluorenylmethyloxycarbonyl-protected *N*-alkyl amino acids by reduction of oxazolidinones. *J. Org. Chem.* **1983**, *48*, 77-81.

S2 Nabika, R.; Suyama, T. L.; Hau, A. M.; Misu, R.; Ohno, H.; Ishmael, J. E.; McPhail, K. L.; Oishi, S.; Fujii, N. Synthesis and biological evaluation of the [D-MeAla[11]]-epimer of coibamide A. *Bioorg. Med. Chem. Lett.* **2015**, *25*, 302-306.

S3 Hou, Z.; Nakanishi, I.; Kinoshita, T.; Takei, Y.; Yasue, M.; Misu, R.; Suzuki, Y.; Nakamura, S.; Kure, T.; Ohno, H.; Murata, K.; Kitaura, K.; Hirasawa, A.; Tsujimoto, G.; Oishi, S.; Fujii, N. Structure-based design of novel potent protein kinase CK2 (CK2) inhibitors with phenyl-azole scaffolds. *J. Med. Chem.* **2012**, *55*, 2899-2903.

S4 Biron, E.; Chatterjee, J.; Kessler, H. Optimized selective N-methylation of peptides on solid support. *J. Pept. Sci.* **2006**, *12*, 213-219.

S5 Pan, Z.; Wu, C.; Wang, W.; Cheng, Z.; Yao, G.; Liu, K.; Li, H.; Fang, L.; Su, W. Total synthesis and stereochemical assignment of gymnopeptides A and B. *Org. Lett.* **2017**, *19*, 4420-4423.

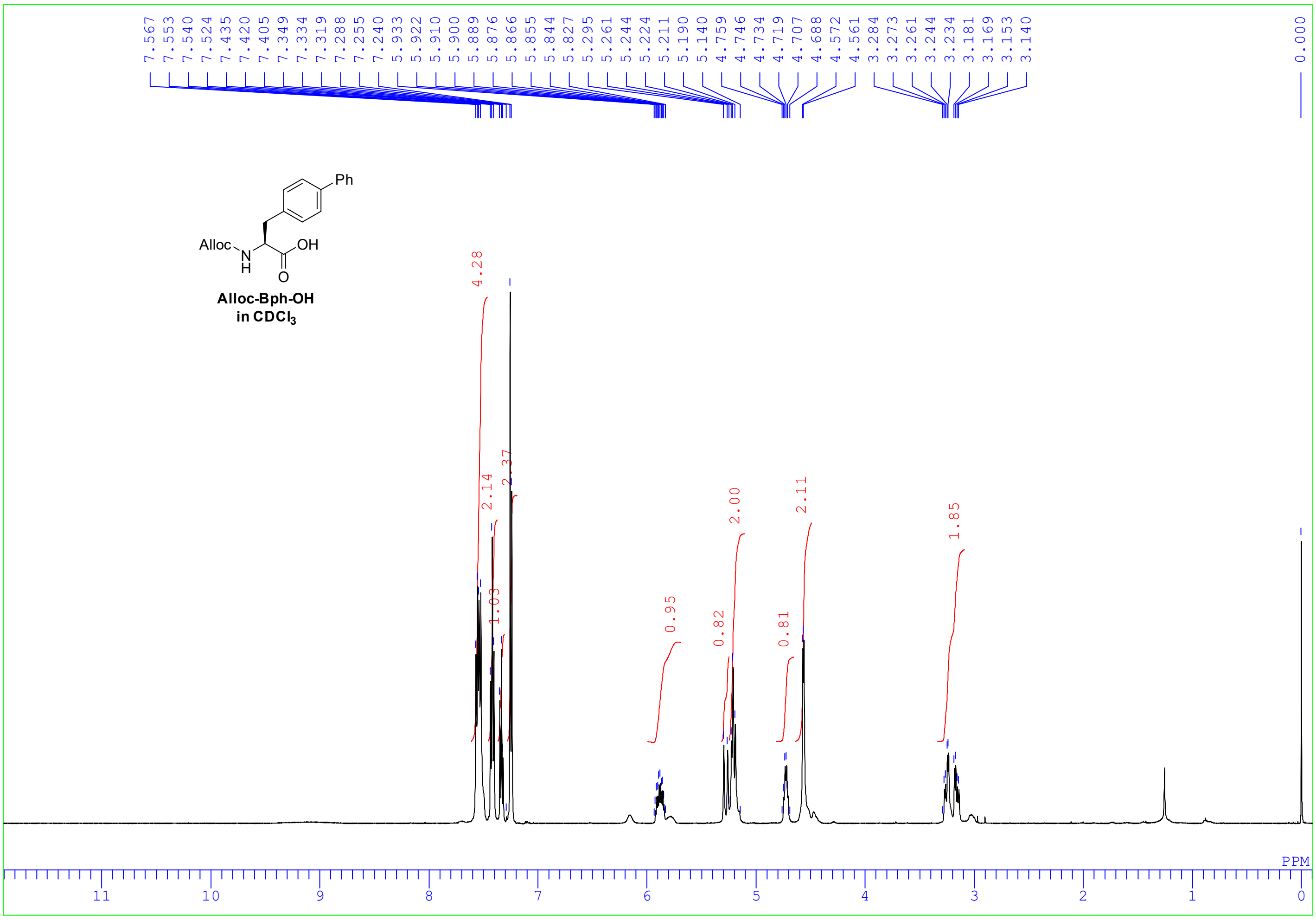

7.567 7.553 7.540 7.524 7.435 7.420 7.405 7.349 7.334 7.319 7.288 7.255 7.240 5.933 5.922 5.910 5.900 5.889 5.876 5.866 5.855 5.844 5.827 5.295 5.261 5.244 5.224 5.211 5.190 5.140 4.759 4.746 4.734 4.719 4.707 4.688 4.572 4.561 3.284 3.273 3.261 3.244 3.234 3.181 3.169 3.153 3.140
0.000
Ph
Alloc
N
H
OH
O
Alloc-Bph-OH
in CDCl3
4.28
2.14
2.37
1.03
0.95
0.82
2.00
0.81
2.11
1.85
PPM
11 10 9 8 7 6 5 4 3 2 1 0

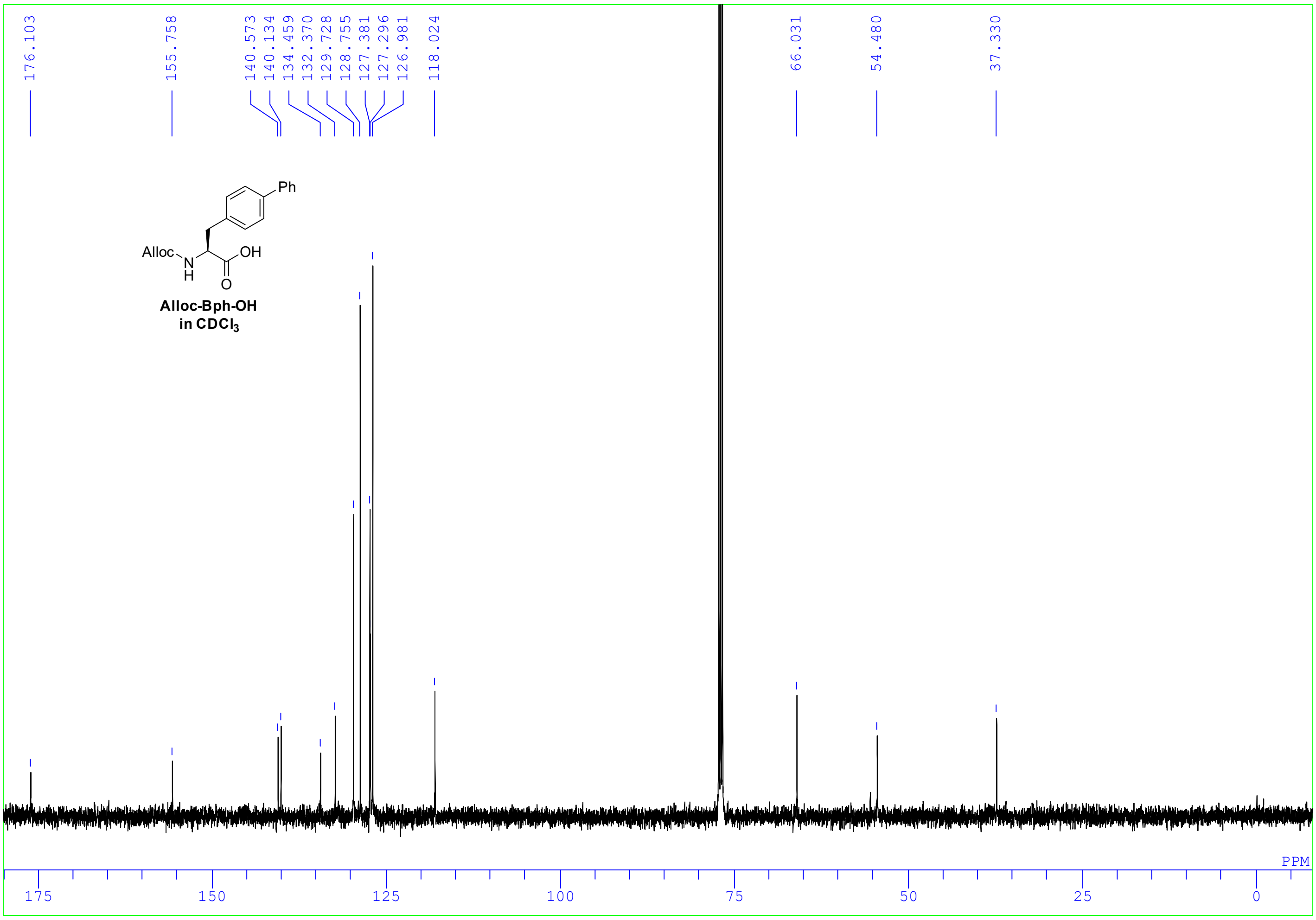

176.103
155.758
140.573 140.134 134.459 132.370 129.728 128.755 127.381 127.296 126.981
118.024
66.031
54.480
37.330
Ph
Alloc
N
H
OH
O
Alloc-Bph-OH
in CDCl3
PPM
175 150 125 100 75 50 25 0

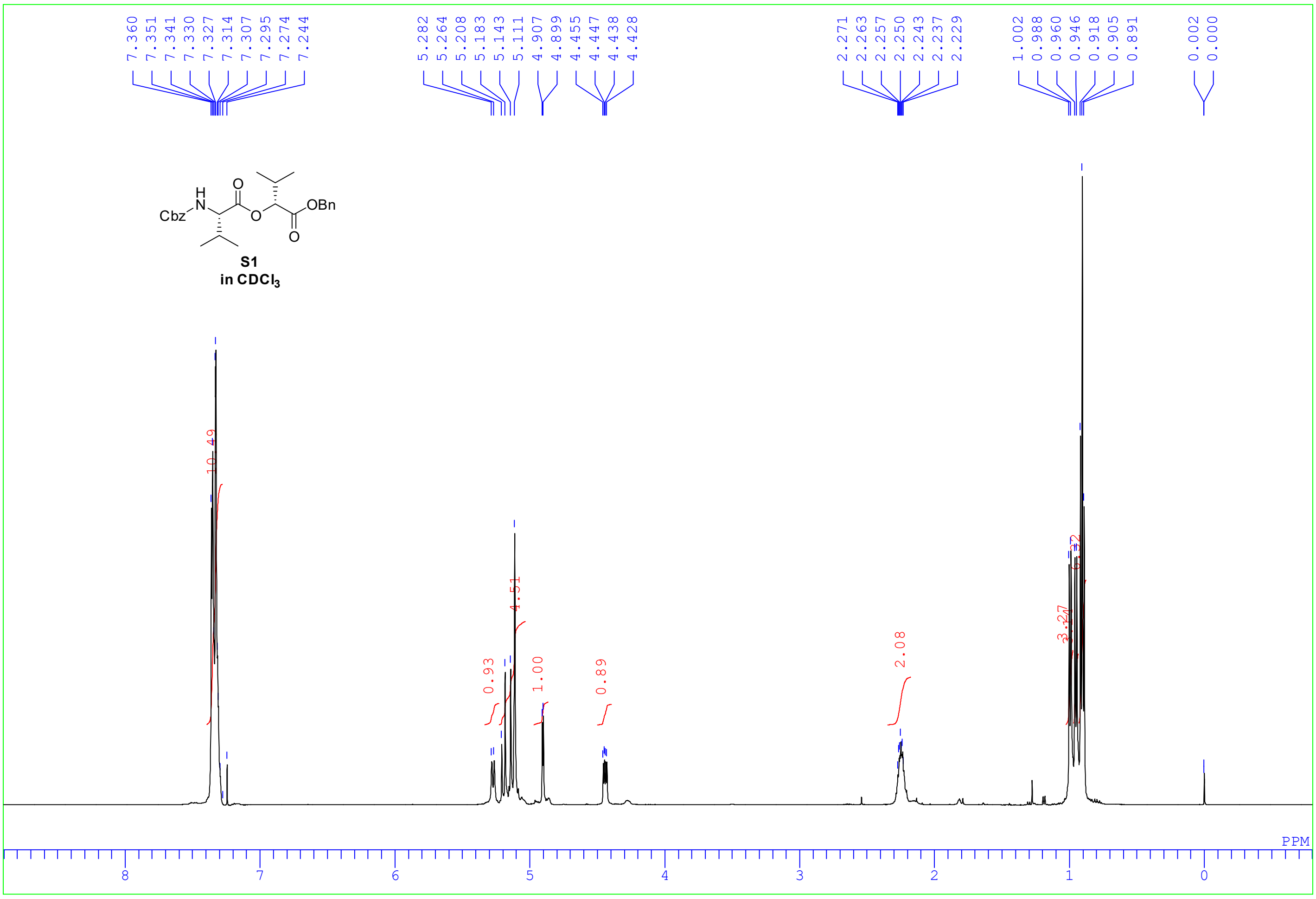

7.360 7.351 7.341 7.330 7.327 7.314 7.307 7.295 7.274 7.244
5.282 5.264 5.208 5.183 5.143 5.111 4.907 4.899 4.455 4.447 4.438 4.428
2.271 2.263 2.257 2.250 2.243 2.237 2.229
1.002 0.988 0.960 0.946 0.918 0.905 0.891
0.002 0.000
Cbz
S1
in CDCl3
10.49
0.93
4.51
1.00
0.89
2.08
3.27
PPM
8
7
6
5
4
3
2
1
0

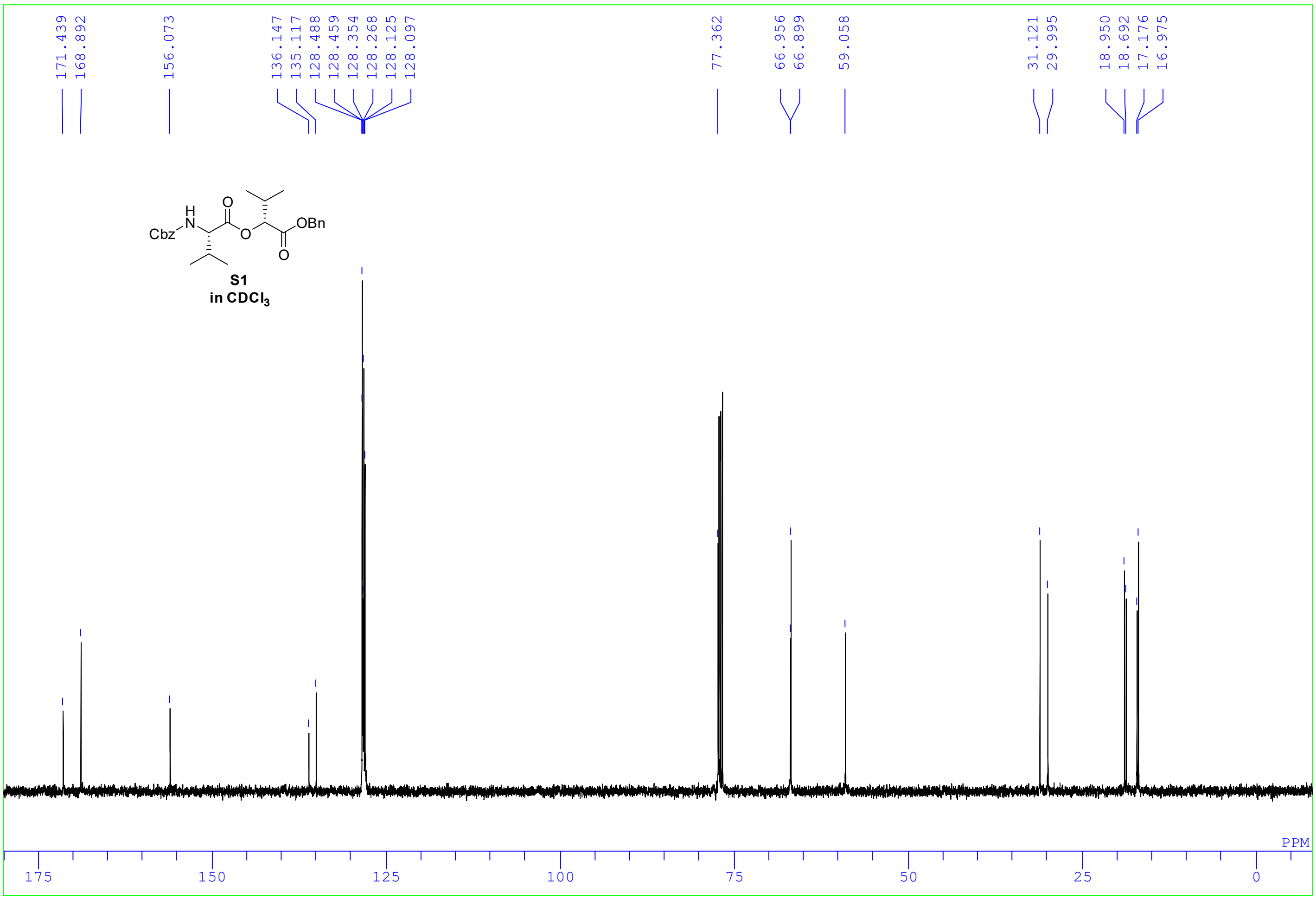

171.439 168.892
156.073
136.147 135.117 128.488 128.459 128.354 128.268 128.125 128.097
77.362
66.956 66.899
59.058
31.121 29.995
18.950 18.692 17.176 16.975
Cbz
S1
in CDCl3
PPM
175
150
125
100
75
50
25
0

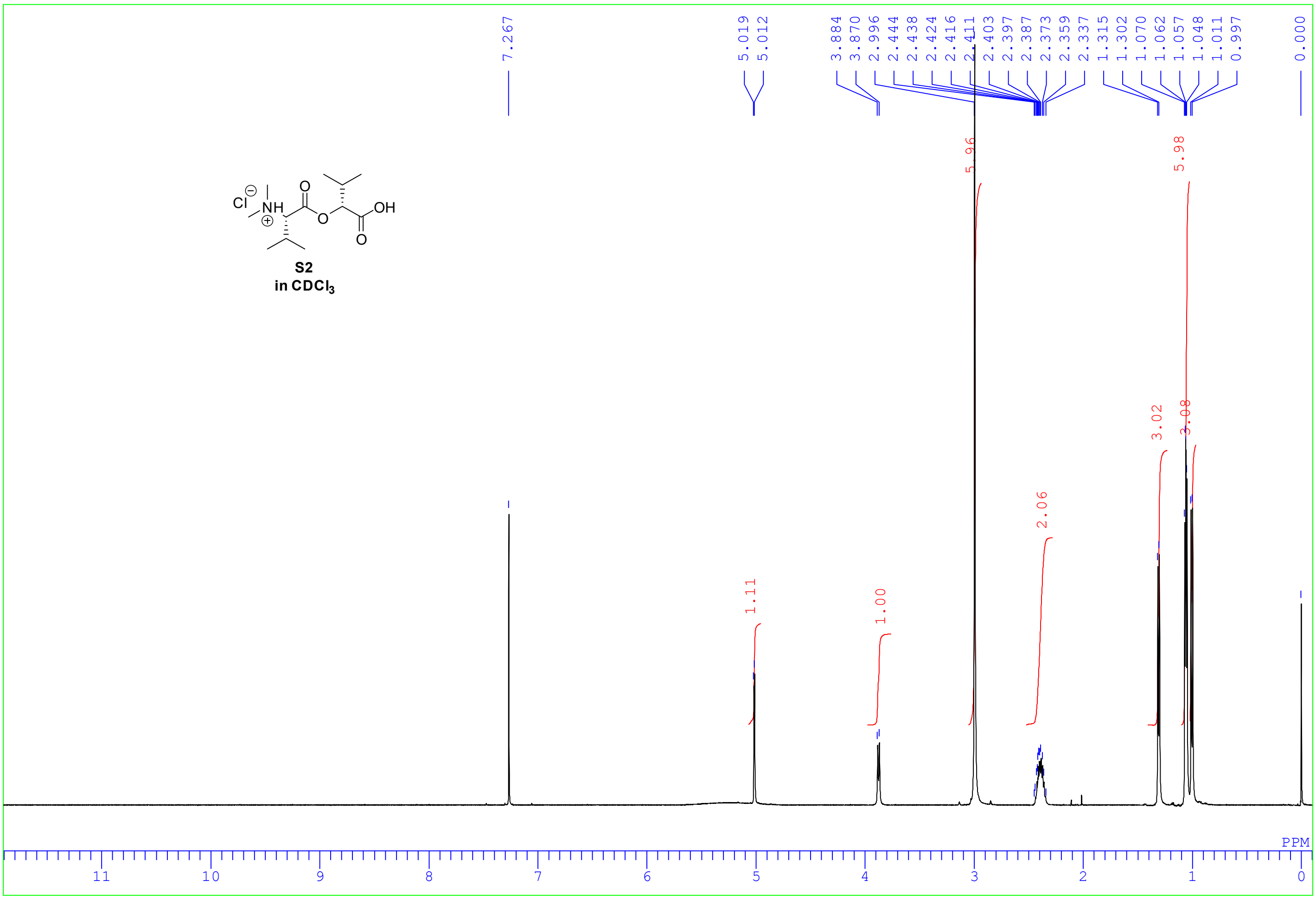
7.267
5.019
5.012
3.884
3.870
2.996
2.444
2.438
2.424
2.416
2.411
2.403
2.397
2.387
2.373
2.359
2.337
1.315
1.302
1.070
1.062
1.057
1.048
1.011
0.997
0.000
1.11
1.00
5.96
2.06
3.02
3.08
5.98
Cl
NH
O
O
OH
O
S2
in $CDCl_3$
PPM
11
10
9
8
7
6
5
4
3
2
1
0

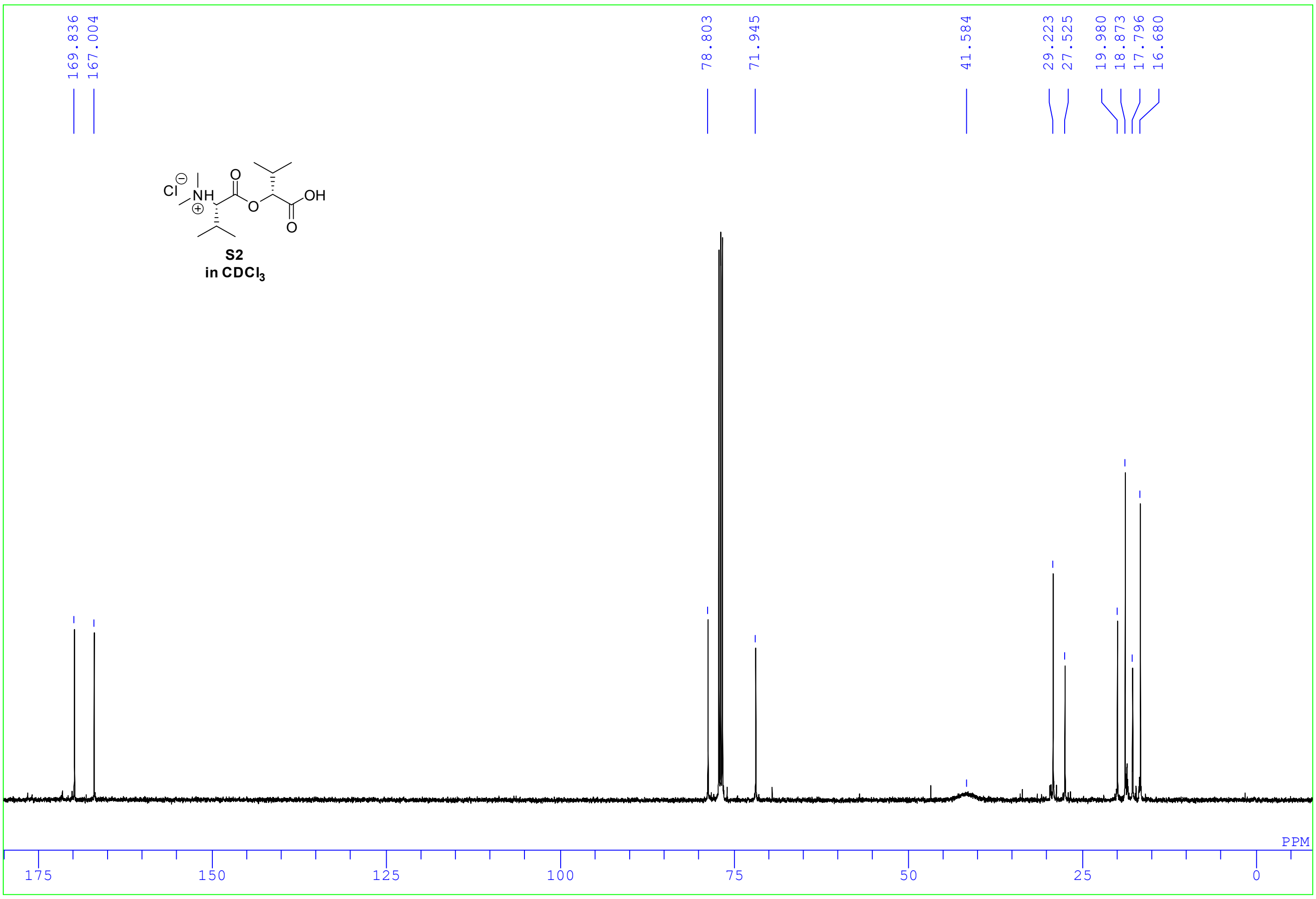
169.836
167.004
78.803
71.945
41.584
29.223
27.525
19.980
18.873
17.796
16.680
Cl
NH
O
O
OH
O
S2
in $CDCl_3$
PPM
175
150
125
100
75
50
25
0

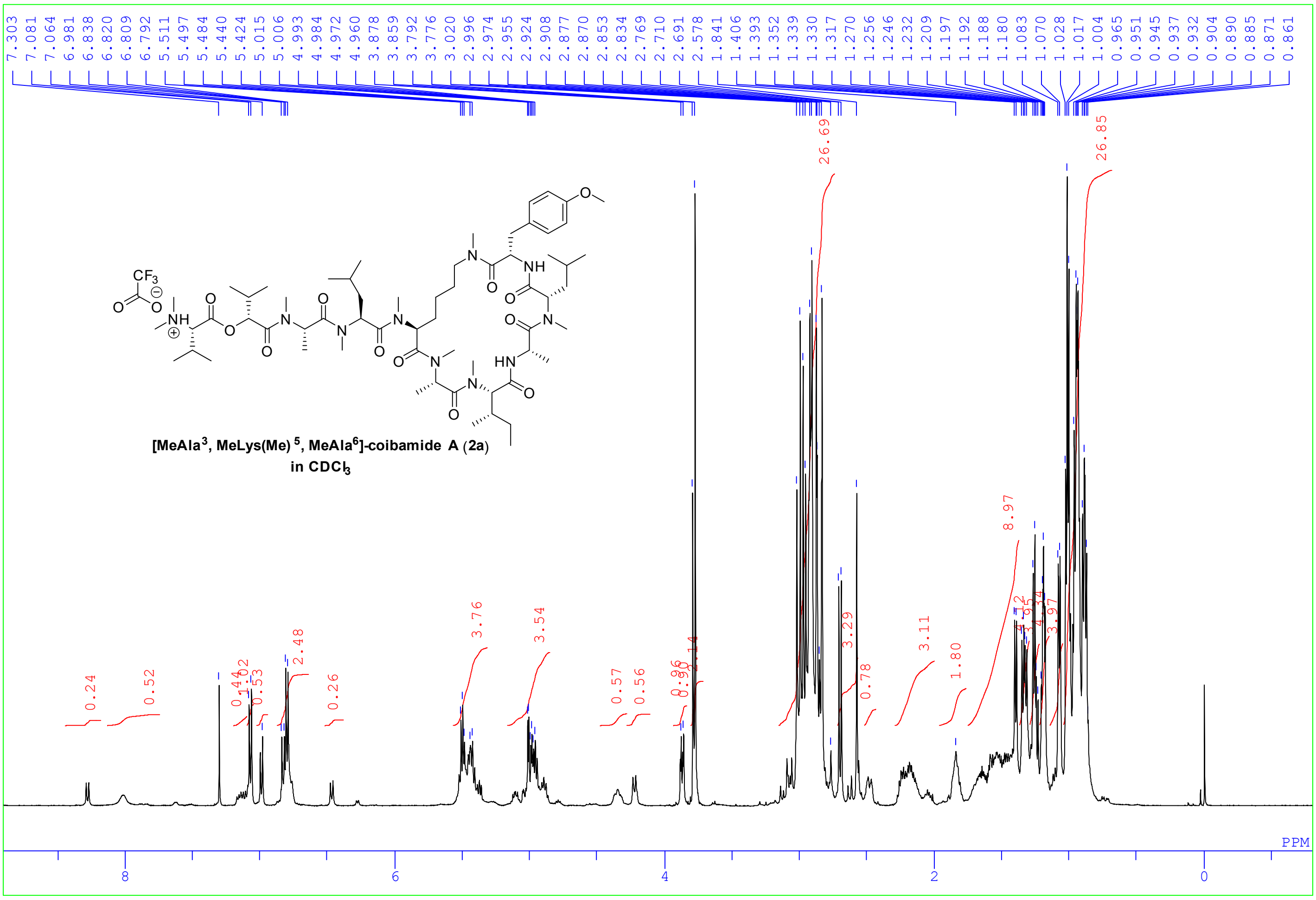
[MeAla3, MeLys(Me)5, MeAla6]-coibamide A (2a)
in CDCl3
PPM

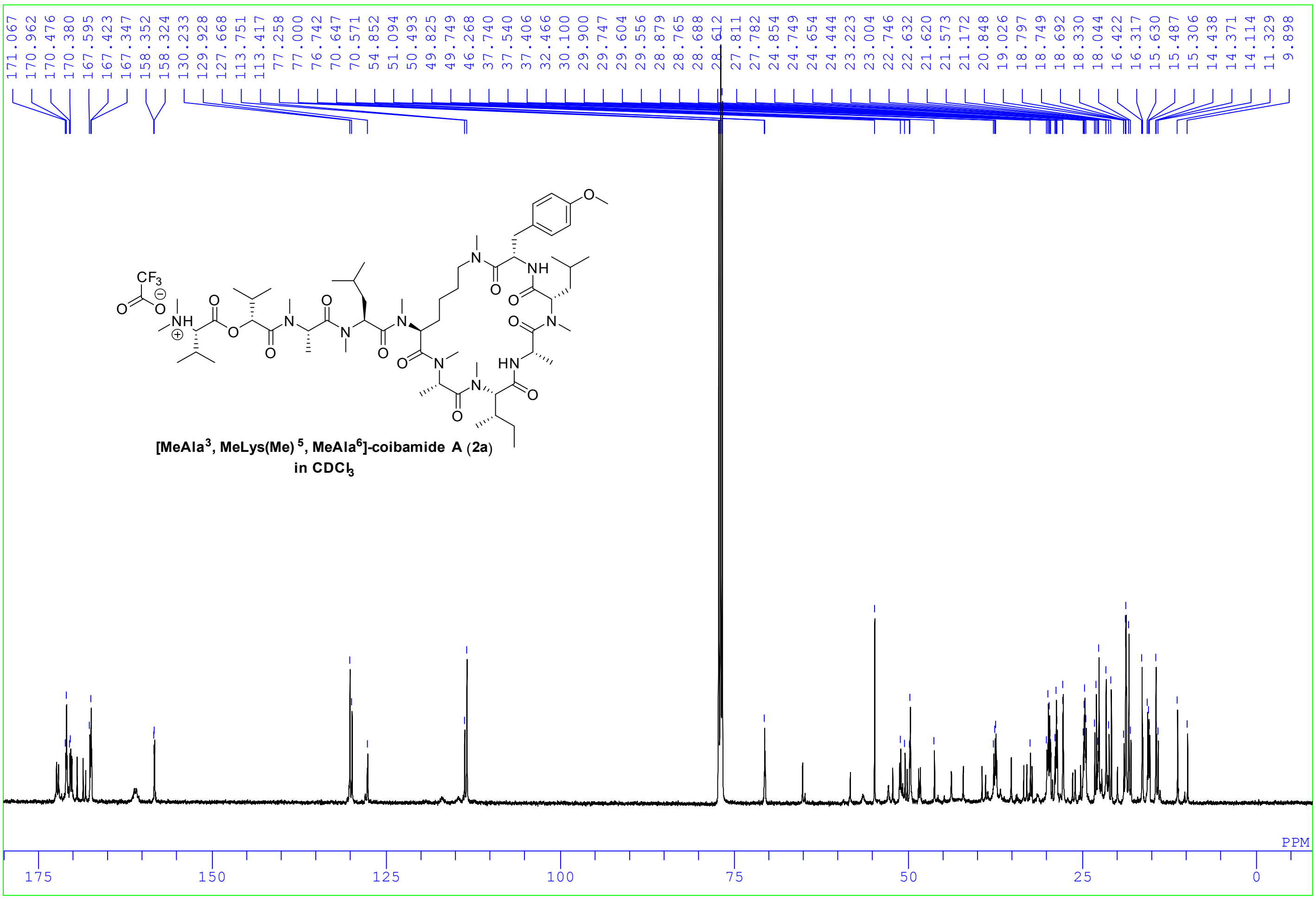
[MeAla3, MeLys(Me)5, MeAla6]-coibamide A (2a)
in CDCl3
PPM

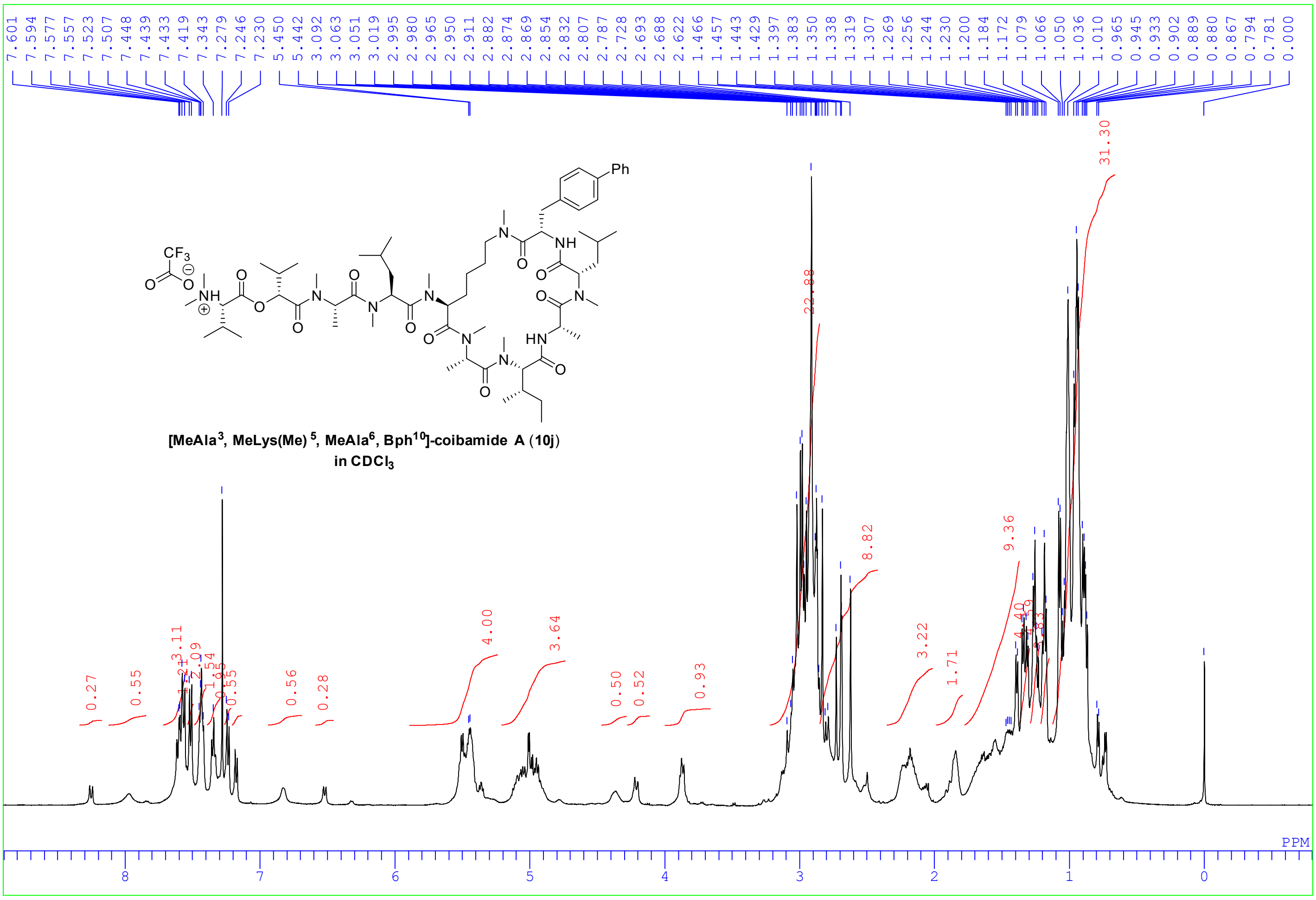
7.601 7.594 7.577 7.557 7.523 7.507 7.448 7.439 7.433 7.419 7.343 7.279 7.246 7.230 5.450 5.442 3.092 3.063 3.051 3.019 2.995 2.980 2.965 2.950 2.911 2.882 2.874 2.869 2.854 2.832 2.807 2.787 2.728 2.693 2.688 2.622 1.466 1.457 1.443 1.429 1.397 1.383 1.350 1.338 1.319 1.307 1.269 1.256 1.244 1.230 1.200 1.184 1.172 1.079 1.066 1.050 1.036 1.010 0.965 0.945 0.933 0.902 0.889 0.880 0.867 0.794 0.781 0.000
[MeAla3, MeLys(Me)5, MeAla6, Bph10]-coibamide A (10j)
in CDCl3
PPM
8
7
6
5
4
3
2
1
0

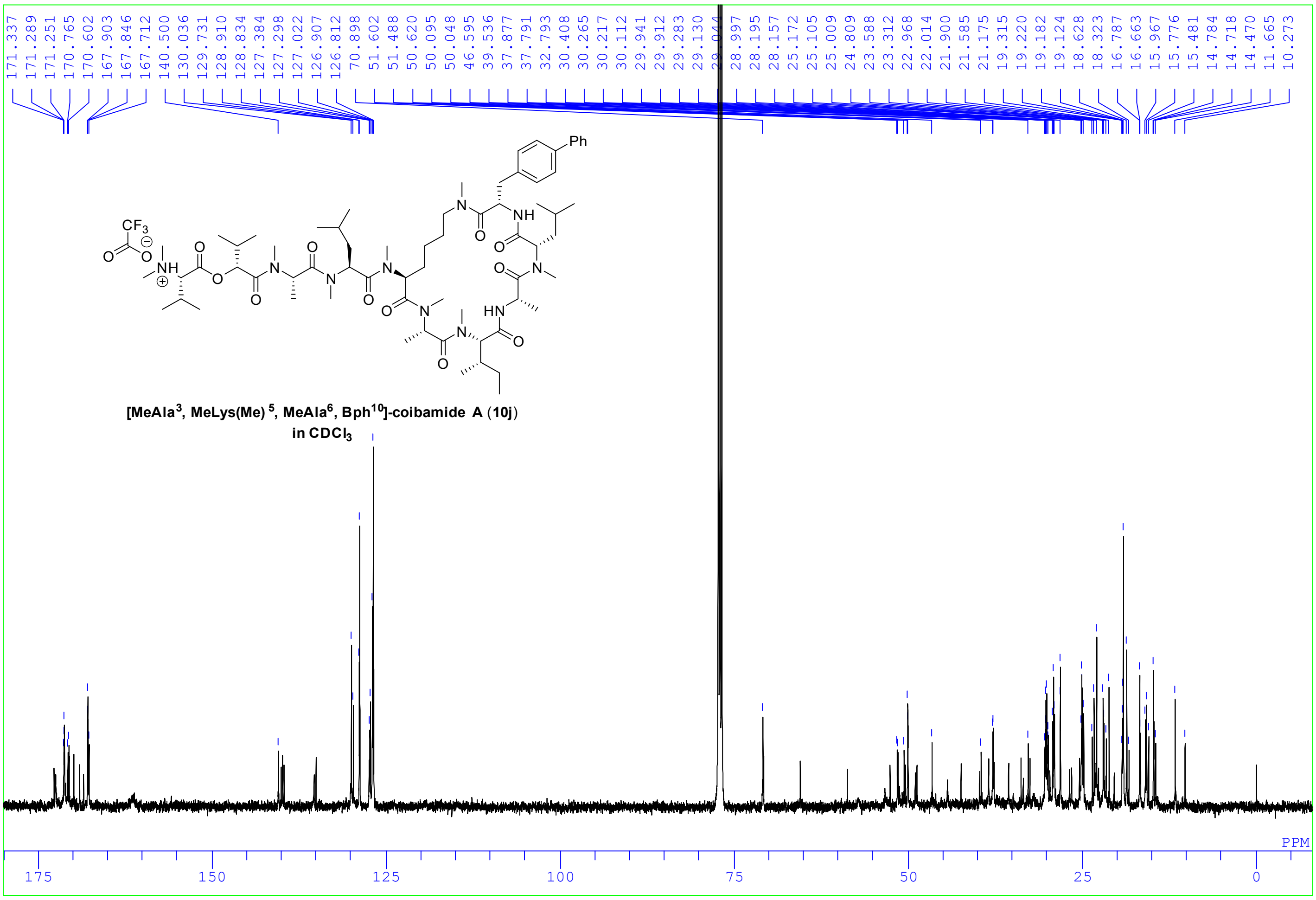
171.337 171.289 171.251 170.765 170.602 167.903 167.846 167.712 140.500 130.036 129.731 128.910 128.834 127.384 127.298 127.022 126.907 126.812 70.898 51.602 51.488 50.620 50.095 50.048 46.595 39.536 37.877 37.791 32.793 30.408 30.265 30.217 30.112 29.941 29.912 29.283 29.130 [illegible] 28.997 28.195 28.157 25.172 25.105 25.009 24.809 23.588 23.312 22.968 22.014 21.900 21.585 21.175 19.315 19.220 19.182 19.124 18.628 18.323 16.787 16.663 15.967 15.776 15.481 14.784 14.718 14.470 11.665 10.273
[MeAla3, MeLys(Me)5, MeAla6, Bph10]-coibamide A (10j)
in CDCl3
PPM
175
150
125
100
75
50
25
0

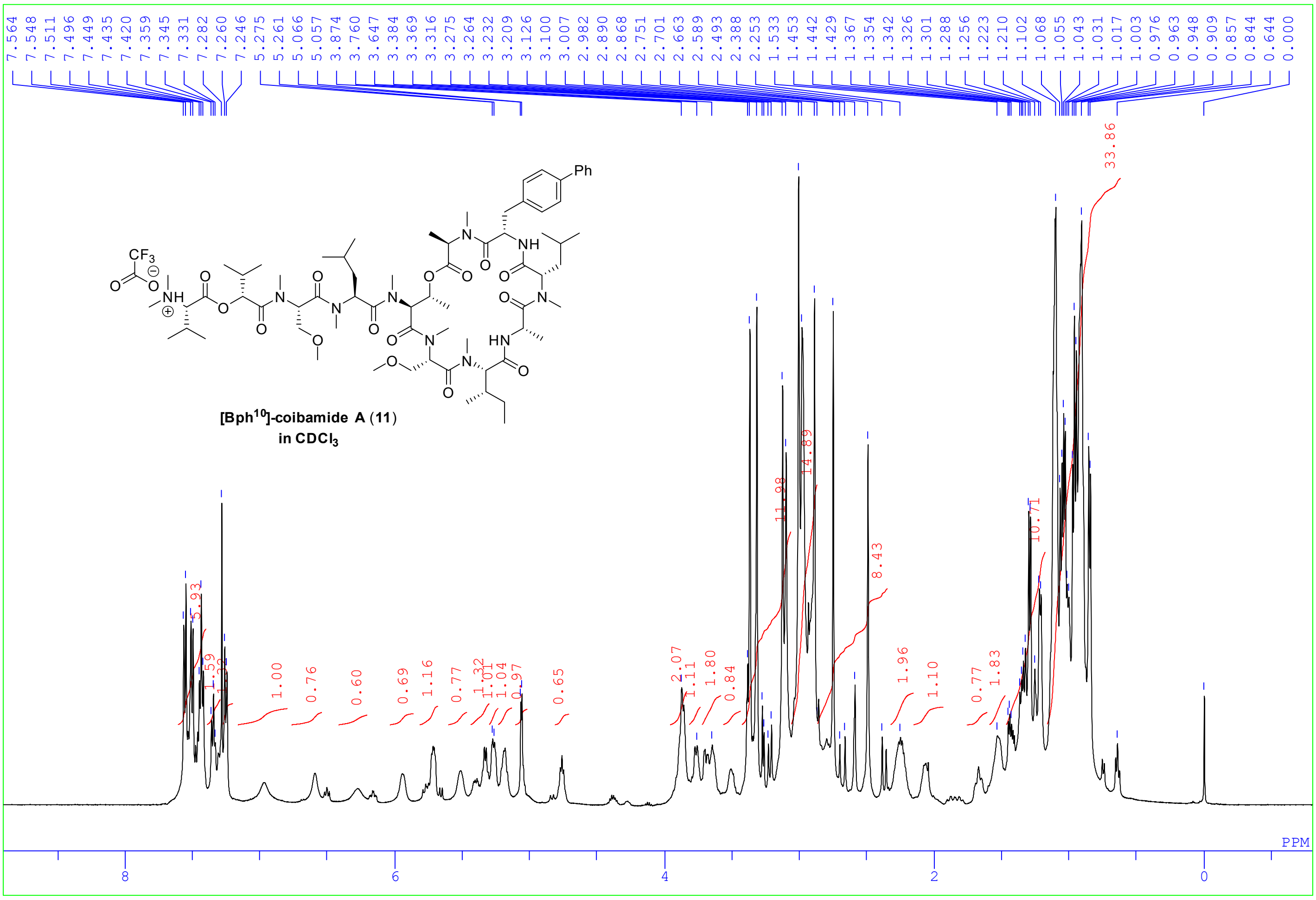
7.564 7.548 7.511 7.496 7.449 7.435 7.420 7.359 7.345 7.331 7.282 7.260 7.246 5.275 5.261 5.066 5.057 3.874 3.760 3.647 3.384 3.369 3.316 3.275 3.264 3.232 3.209 3.126 3.100 3.007 2.982 2.890 2.868 2.751 2.701 2.663 2.589 2.493 2.388 2.253 1.533 1.453 1.442 1.429 1.367 1.354 1.342 1.326 1.301 1.288 1.256 1.223 1.210 1.102 1.068 1.055 1.043 1.031 1.017 1.003 0.976 0.963 0.948 0.909 0.857 0.844 0.644 0.000
Ph
CF3
NH
[Bph10]-coibamide A (11)
in CDCl3
5.93
1.00
0.76
0.60
0.69
1.16
0.77
1.32
1.01
1.04
0.97
0.65
2.07
1.11
1.80
0.84
11.98
14.89
8.43
1.96
1.10
0.77
1.83
10.71
33.86
PPM
8
6
4
2
0

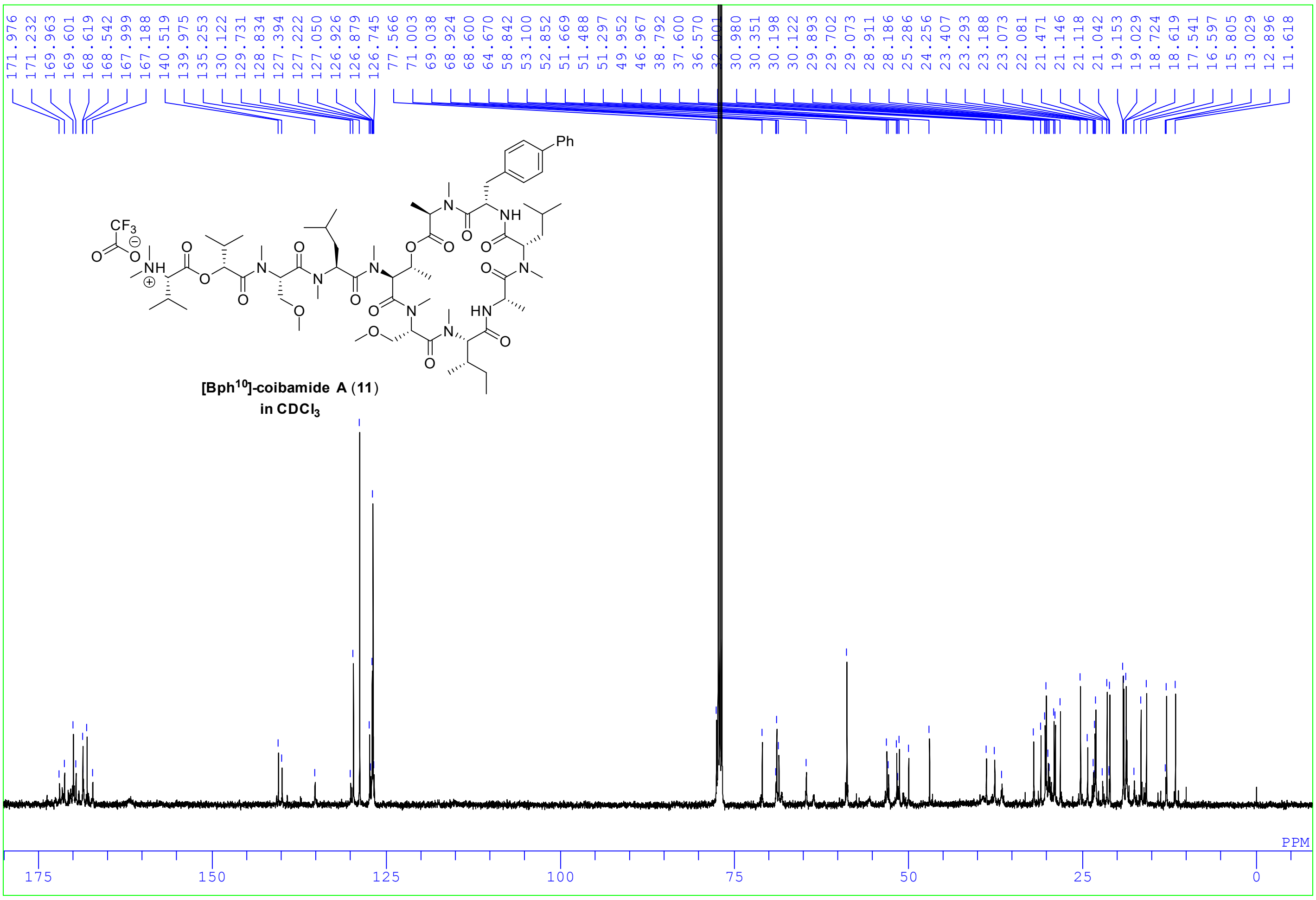
171.976 171.232 169.963 169.601 168.619 168.542 167.999 167.188 140.519 139.975 135.253 130.122 129.731 128.834 127.394 127.222 127.050 126.926 126.879 126.745 77.566 71.003 69.038 68.924 68.600 64.670 58.842 53.100 52.852 51.669 51.488 51.297 49.952 46.967 38.792 37.600 36.570 30.980 30.351 30.198 30.122 29.893 29.702 29.073 28.911 28.186 25.286 24.256 23.407 23.293 23.188 23.073 22.081 21.471 21.146 21.118 21.042 19.153 19.029 18.724 18.619 17.541 16.597 15.805 13.029 12.896 11.618
Ph
CF3
NH
[Bph10]-coibamide A (11)
in CDCl3
PPM
175
150
125
100
75
50
25
0

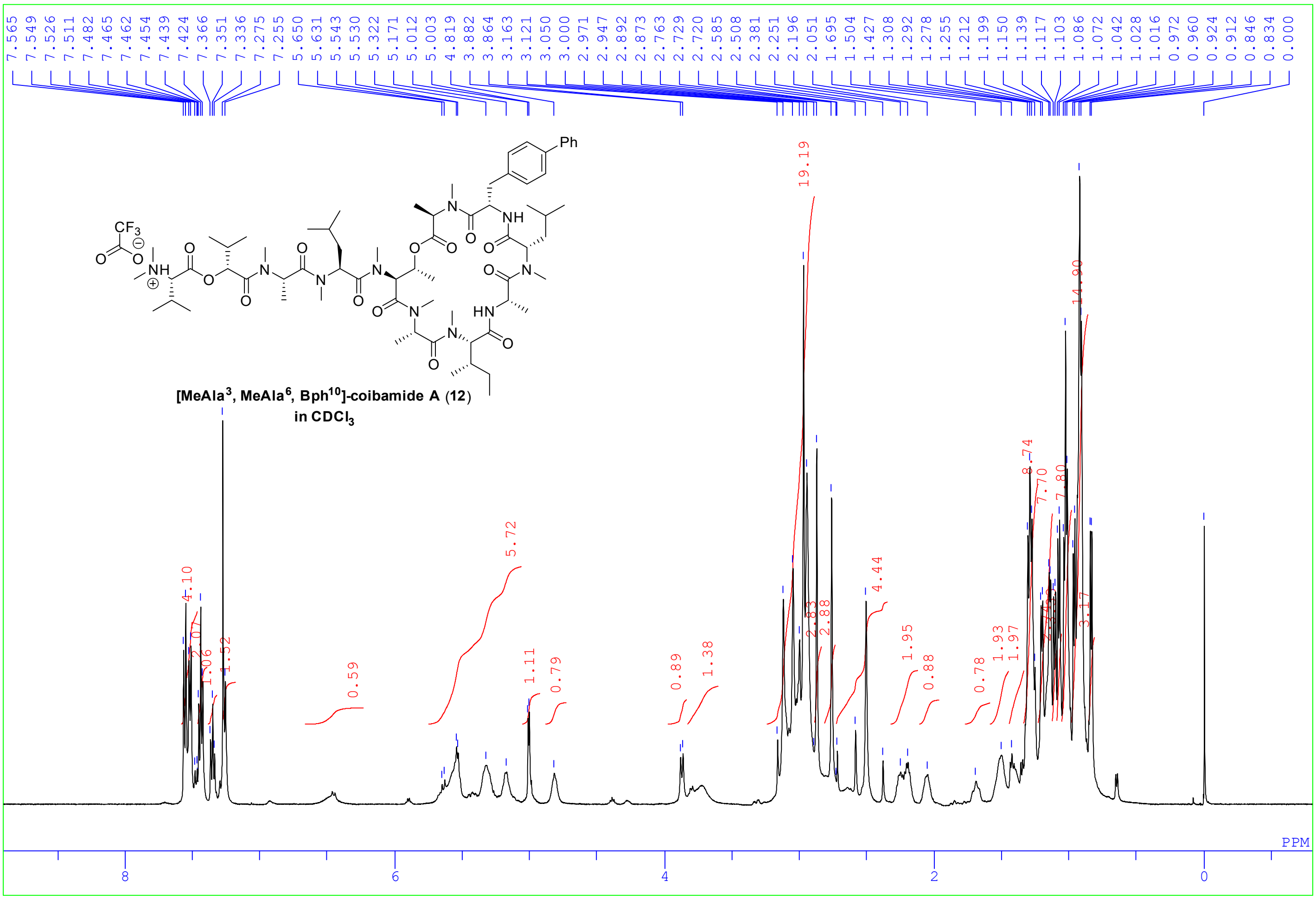
[MeAla3, MeAla6, Bph10]-coibamide A (12)
in CDCl3
PPM

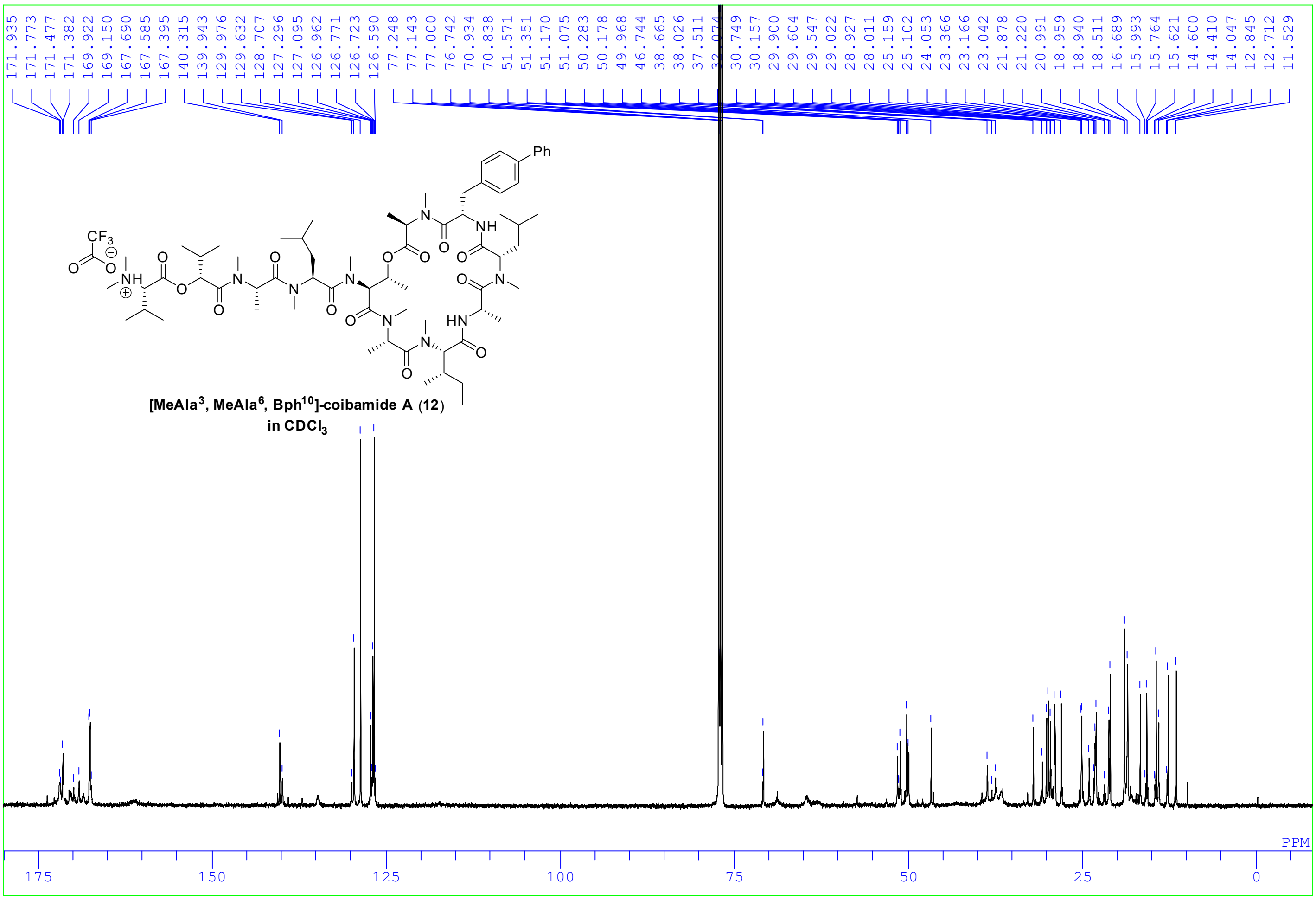
[MeAla3, MeAla6, Bph10]-coibamide A (12)
in CDCl3
PPM
